# Characterization and Vaccine Potential of Membrane Vesicles Produced by Francisella noatunensis subsp. orientalis in an Adult Zebrafish Model

**DOI:** 10.1128/CVI.00557-16

**Published:** 2017-05-05

**Authors:** Leidy Lagos, Julia I. Tandberg, Urska Repnik, Preben Boysen, Erik Ropstad, Deepa Varkey, Ian T. Paulsen, Hanne C. Winther-Larsen

**Affiliations:** a Center of Integrative Microbial Evolution and Department of Pharmaceutical Biosciences, School of Pharmacy, Faculty of Mathematics and Natural Science, University of Oslo, Oslo, Norway; b Department of Biosciences, Faculty of Mathematics and Natural Science, University of Oslo, Oslo, Norway; c Department of Food Safety & Infection Biology, Faculty of Veterinary Medicine and Biosciences, Norwegian University of Life Sciences, Oslo, Norway; d Department of Production Animal Clinical Sciences, Norwegian University of Live Sciences, NMBU-School of Veterinary Science, Oslo, Norway; e Department of Chemistry and Biomolecular Sciences, Macquarie University, Sydney, New South Wales, Australia; University of Maryland School of Medicine

**Keywords:** Francisella noatunensis subsp. orientalis, membrane vesicles, MVs, vaccine, francisellosis, zebrafish, immune response, fish pathogens, immunization, immunology

## Abstract

Vaccine development against extracellular bacteria has been important for the sustainability of the aquaculture industry. In contrast, infections with intracellular pathogens remain largely an unresolved problem. Francisella noatunensis subsp. orientalis is a Gram-negative, facultative intracellular bacterium that causes the disease francisellosis in fish. Francisellosis is commonly characterized as a chronic granulomatous disease with high morbidity and can result in high mortality depending on the host. In this study, we explored the potential of bacterial membrane vesicles (MVs) as a vaccine agent against F. noatunensis subsp. orientalis. Bacterial MVs are spherical structures naturally released from the membrane of bacteria and are often enriched with selected bacterial components such as toxins and signaling molecules. MVs were isolated from broth-cultured F. noatunensis subsp. orientalis in the present work, and proteomic analysis by mass spectrometry revealed that MVs contained a variety of immunogenic factors, including the intracellular growth proteins IglC and IglB, known to be part of a Francisella pathogenicity island (FPI), as well as outer membrane protein OmpA, chaperonin GroEL, and chaperone ClpB. By using flow cytometry and electron microscopy, we observed that F. noatunensis subsp. orientalis mainly infects myelomonocytic cells, both *in vivo* and *in vitro*. Immunization with MVs isolated from F. noatunensis subsp. orientalis protects zebrafish from subsequent challenge with a lethal dose of F. noatunensis subsp. orientalis. To determine if MVs induce a typical acute inflammatory response, mRNA expression levels were assessed by quantitative real-time PCR. Expression of *tnfa*, *il1b*, and *ifng*, as well as *mhcii*, *mpeg1.1*, and *ighm*, was upregulated, thus confirming the immunogenic properties of F. noatunensis subsp. orientalis-derived MVs.

## INTRODUCTION

Francisella species are nonmotile, pleomorphic, Gram-negative, strictly aerobic, facultative intracellular coccobacilli. The genus includes species capable of causing disease in a range of hosts, including humans (Francisella tularensis and F. philomiragia) and fish (F. noatunensis) (1). F. noatunensis is the causative agent of piscine francisellosis in a number of fish species (2–4) and consists of two subspecies, which appear to be adapted to the different body temperatures of their respective host organisms: F. noatunensis subsp. orientalis causes disease in warm-water fish, such as tilapia (Oreochromis niloticus), while F. noatunensis subsp. noatunensis causes disease in fish living in colder waters, such as cod (Gadus morhua) and Atlantic salmon (Salmo salar) (3, 4). Infected fish develop nonspecific clinical signs and multifocal granulomatous lesions and can suffer high mortality rates (5).

Introducing vaccination to fish farming has decreased the use of antibiotics, while the production of fish has increased. Vaccination has become the best prevention strategy to combat disease in high-density finfish aquaculture. Most of the fish vaccines currently on the market are formulations of inactivated bacteria or viruses using mineral oil as an adjuvant and are administered intraperitoneally (i.p.). The desired immune response to a vaccine is the production of antibodies and/or generation of cytotoxic T cells (6). In contrast to extracellular bacteria, protection against intracellular bacteria usually also requires a cellular immune response. No commercial vaccine against fish francisellosis is currently available; however, attempts using attenuated mutant strains of F. noatunensis subsp. orientalis have provided promising results in tilapia (7).

Bacterial pathogens have developed numerous strategies to deliver virulence factors to the eukaryotic host cell; one of these strategies is the release of membrane vesicles (MVs) (8). MVs are spherical, with a diameter of 10 to 300 nm. Proteomic and biochemical characterization has revealed that the vesicles contain a variety of bacterial components, including periplasmic and outer membrane (OM) proteins as well as lipopolysaccharides (LPS), DNA, RNA, and cytoplasmic proteins (9, 10). MVs also contain several important immunogenic factors, such as toxins (11), chaperons, and active enzymes (12). The secretion of MVs can be increased during stress and environmental changes. These include treatment with membrane-active antibiotics, nutrient depletion, temperature alteration, and chemical exposure (13, 14), all factors that a bacterium may encounter both in its natural environment and within a host. Numerous studies have shown that several species of the genus Francisella, including F. tularensis subsp. novicida (15), F. philomiragia (12), and F. noatunensis subsp. noatunensis (16), are able to produce MVs. Isolated MVs have the capability to induce a protective immune response to the infectious agent and have therefore been investigated and used as vaccines (17). Membrane vesicle vaccines in mice induce protective responses to subsequent challenge with F. tularensis subsp. novicida (8), and they induce the release of proinflammatory cytokines by macrophages *in vitro* (18). Due to the immunogenic characteristics of these structures, MV-based vaccines have been successfully used against meningococcal disease in humans (19). The use of MVs as vaccines has also been investigated in several fish species, such as Edwardsiella tarda in olive flounder (Paralichthys olivaceus) (20), Flavobacterium psychrophilum in rainbow trout (Oncorhynchus mykiss) (21), and F. noatunensis subsp. noatunensis in zebrafish (Danio rerio) (16).

In recent years, zebrafish has proven to be a unique vertebrate model for the study of leukocyte subsets, immune cell migration, and host-pathogen interactions. Several research groups have attempted to identify pathogens that can infect and cause disease in zebrafish, including Streptococcus pyogenes, Streptococcus iniae (22), Mycobacterium marinum (23), and Edwarsiella tarda (24), showing zebrafish to be a valuable tool for the study of immunity. Both adult zebrafish and zebrafish larvae were found to be suitable models for studies of Francisella infection (16, 25–27). Adult zebrafish infected with F. noatunensis subsp. orientalis undergo an acute disease process, succumbing to infection in a dose-dependent manner (26). In zebrafish larvae, F. noatunensis subsp. orientalis mainly infects macrophages, inducing a robust proinflammatory immune response, dominated by increased transcription of tumor necrosis factor alpha (*tnfa*) and interleukin-1β (*il1b*) genes (27). Considering that larvae do not possess adaptive immunity, it was of interest to dissect the main target cells in adult zebrafish, which show innate as well as adaptive immunity. By demonstrating the effect of F. noatunensis subsp. orientalis on zebrafish cells, as measured by flow cytometry and electron microscopy (EM), we confirmed zebrafish to be a valuable infection model for the study of francisellosis. Further, we examined the protein content of MVs from F. noatunensis subsp. orientalis and its use as an acellular vaccine against francisellosis.

## RESULTS

### F. noatunensis subsp. orientalis is internalized by zebrafish leukocytes *in vitro*.

**FIG 1:**
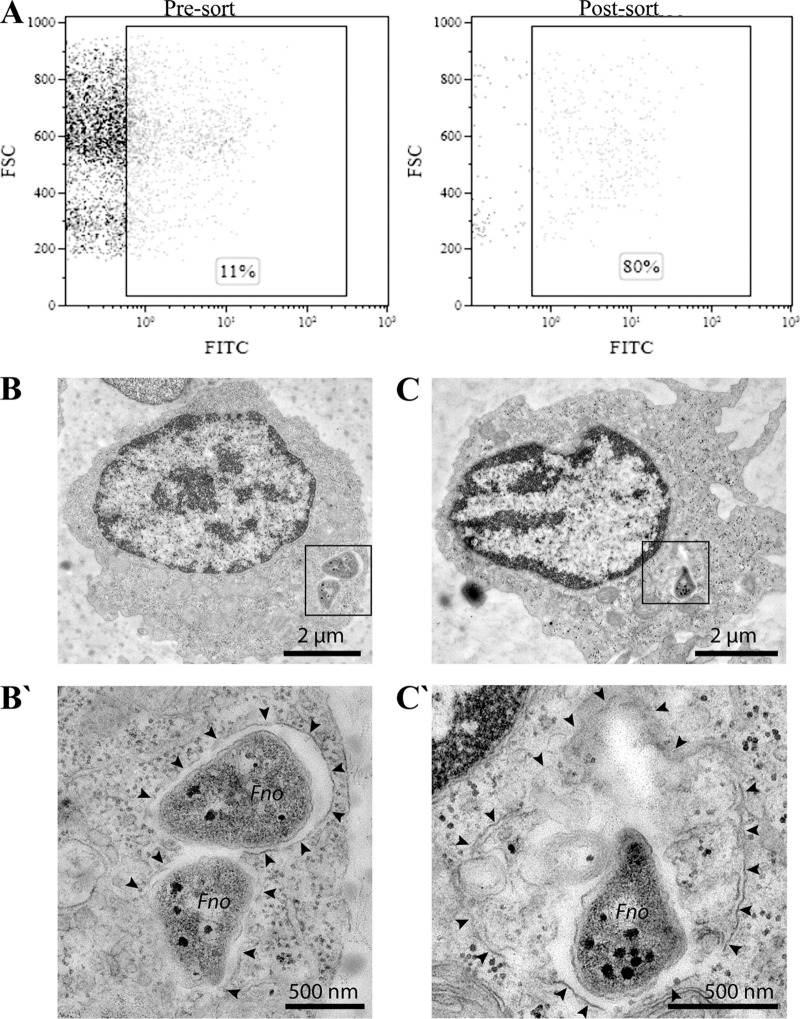
F. noatunensis subsp. orientalis uptake by zebrafish leukocytes *in vitro*. (A) Representative dot plot before (Pre-sort) and after (Post-sort) cell sorting of leukocytes harvested from adult zebrafish kidney incubated for 1 h with *Fno*-FITC. Bacterial uptake was quantified by flow cytometry as the percentage of cells with increased FITC fluorescence. (B to C′) Ultrastructural TEM analysis of zebrafish kidney leukocytes with internalized F. noatunensis subsp. orientalis (*Fno*) after 1 h of incubation. In macrophages (B and C), bacteria reside mostly in tight phagosomes (B′) and only rarely in larger phagolysosomes (C′) with additional luminal cargo. Black arrowheads indicate phagosomal/phagolysosomal membrane. Bars, 2 μm (B to C) and 500 nm (B′ to C′).

To identify the main type of immune cells infected by F. noatunensis subsp. orientalis, kidney leukocytes isolated from adult zebrafish were cultivated *in vitro* in the presence of F. noatunensis subsp. orientalis labeled with fluorescein isothiocyanate (FITC), and the uptake of bacteria was analyzed by flow cytometry after 1 h. Due to the lack of antibodies against different immune cells in fish, hematopoietic linages were identified on the basis of forward scatter (FSC) and side scatter (SSC) of light as shown by Traver et al. (28) (see Fig. S1 in the supplemental material). The location of the infected cells in the FSC/SSC dot plot shows that mainly myelomonocytic cells were infected (rectangular insert, Fig. 1A [Pre-sort]) and that 11% of these cells were able to incorporate F. noatunensis subsp. orientalis FITC (*Fno*-FITC). After sorting, the population containing F. noatunensis subsp. orientalis was enriched to up to 80% purity (Fig. 1A [Post-sort]) and its ultrastructural features were examined by transmission electron microscopy (TEM). Some of the cells containing F. noatunensis subsp. orientalis exhibited characteristics of macrophages, namely, low nucleus-to-cytoplasm ratios and branched projections emanating in all directions from the cell body, with agranular cytoplasm (29). At 1 h after the leukocyte incubation with F. noatunensis subsp. orientalis (Fig. 1B and C), bacteria were membrane bound and resided mostly in tight phagosomes/vacuoles with little or no additional luminal cargo (Fig. 1B′). The limiting membrane was often poorly preserved or only partially visible. Some bacteria were observed in larger phagolysosomes with abundant luminal cargo (Fig. 1C′). Similar results have been observed for other species of Francisella, such as F. tularensis, inside human macrophage-derived cell lines (30) and for F. noatunensis subsp. noatunensis in Dictyostelium discoideum amoebas (25). We also noted bacteria residing in neutrophil phagosomes, in which many of them appeared damaged and degraded even at this early time point (data not shown). Thus, our results are similar to those obtained on zebrafish larvae, where macrophages are the main host cell relevant for the infection, whereas neutrophils are attracted to and take up F. noatunensis subsp. orientalis under conditions of solid surface support rather than when infected via the circulation system (27).

### The presence of F. noatunensis subsp. orientalis reduces the number of myelomonocytic cells but does not result in neutrophil infection *in vivo*.

**FIG 2:**
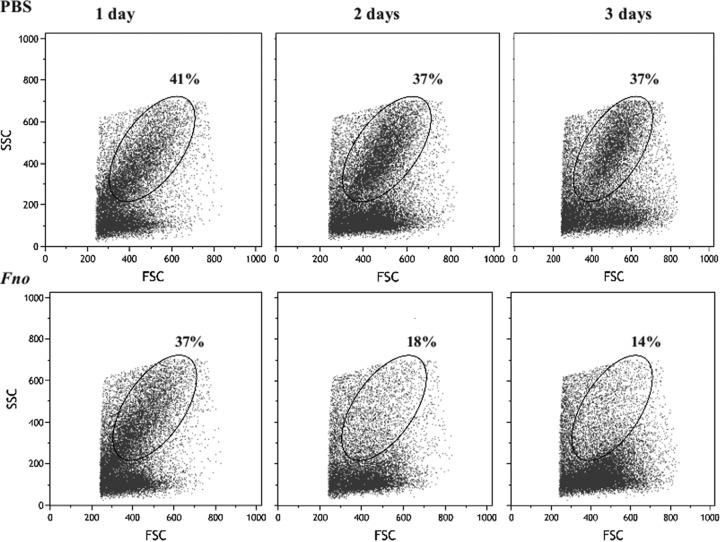
F. noatunensis subsp. orientalis infection reduces the number of myelomonocytic cells *in vivo*. Adult wild-type zebrafish were challenged with PBS (upper panels) or 10^6^ CFU F. noatunensis subsp. orientalis (*Fno*; lower panels). Kidneys were isolated 1, 2, or 3 days after challenge, and the main hematopoietic linages were observed using light-scattering gating by flow cytometry. The ovals represent the myelomonocytic population. Values are representative of three biological replicates.

**FIG 3:**
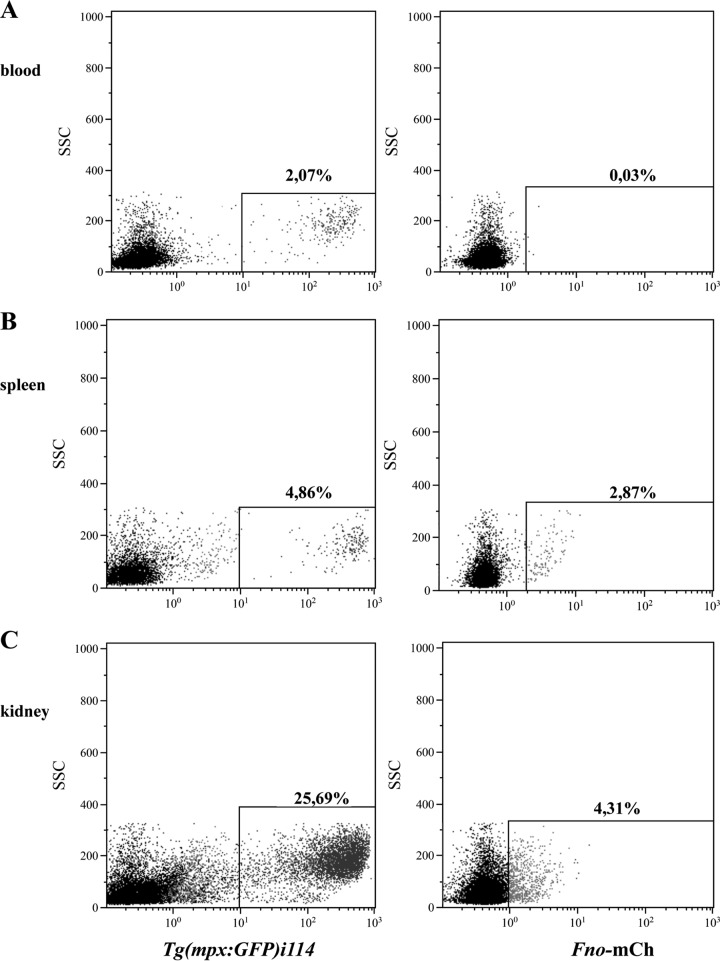
Incorporation of F. noatunensis subsp. orientalis analyzed *in vivo* by flow cytometry. Transgenic adult zebrafish [*Tg*(*mpx:GFP*)^*i114*^] were challenged with 10^6^ CFU *Fno*-mCherry (*Fno*-mCh). Blood (A), spleen (B), and kidney (C) leukocytes were isolated at 2 h after infection and analyzed by flow cytometry. Dot plots show the percentages of neutrophil (left column) and the percentages of cells able to internalize F. noatunensis subsp. orientalis (right column). Values are representative of experiments performed with three replicates.

As described by others (28), kidney cells isolated from adult zebrafish contain two major leukocyte populations identified by light scattering, excluding aggregates and red cells: one corresponding to myelomonocytic cells and the other to lymphoid/precursor cells. F. noatunensis subsp. orientalis infection reduced the number of cells belonging to the myelomonocytic population, which includes monocytes, dendritic cells, macrophages, and neutrophils (Fig. 2). The data show that the proportion of the myelomonocytic population in kidney of infected fish was reduced from 37% 1 day after infection to 14% 3 days after infection compared with control animals receiving phosphate-buffered saline (PBS), in which myelomonocytic cells represented 37% to 41% of kidney leukocytes during the 3 days after challenge. Unfortunately, we were not able to use the macrophage transgenic zebrafish *mpeg1.1* gene, as presented in a previous report from our group (27). The reason is that macrophage-specific reporter *mpeg1.1* is active only in the embryo and the larval state, precluding its use for the study of macrophages in adult fish. However, fish of a transgenic zebrafish line expressing enhanced green fluorescent protein (GFP) in neutrophilic granulocytes [*Tg*(*mpx:GFP*)^*i114*^] were infected with F. noatunensis subsp. orientalis transformed with mCherry-expressing plasmid pKK289Km/*mCherry* (*Fno*-mCherry), and the infection was followed *in vivo*. Figure 3 shows the expression of *Tg*(*mpx:GFP*)^*i114*^ protein and *Fno*-mCherry at 2 h after infection in different organs. The fraction of neutrophils in the leukocyte population of kidney (25.69%) was significantly higher than that in blood or spleen (2.07% or 4.86%, respectively). We were not able to detect *Fno*-mCherry in blood at any time point. However, after 2 h of infection, 2.87% of splenocytes contained *Fno*-mCherry. Interestingly, the cells that were infected with F. noatunensis subsp. orientalis were GFP negative. This might indicate that *Fno*-mCherry was not taken up by neutrophil or that *Fno*-mCherry was degraded by the neutrophil population within the 2-h period. This is similar to results seen with the larval state, in which macrophages were shown to be preferentially infected by F. noatunensis subsp. orientalis injected into the blood, while neutrophils were more active under conditions of solid support (27). The main organ affected by F. noatunensis subsp. orientalis infection was kidney, with 4.31% of leukocytes infected with F. noatunensis subsp. orientalis at 2 h after infection, and this rate of infection was maintained also at 24 and 48 h after infection (Fig. S2), but the infection seems not to have affected the neutrophils. We can conclude that F. noatunensis subsp. orientalis infects mainly leukocytes in kidney and induces a reduction of the myelomonocytic population.

### Isolation and characterization of MVs from F. noatunensis subsp. orientalis.

**FIG 4:**
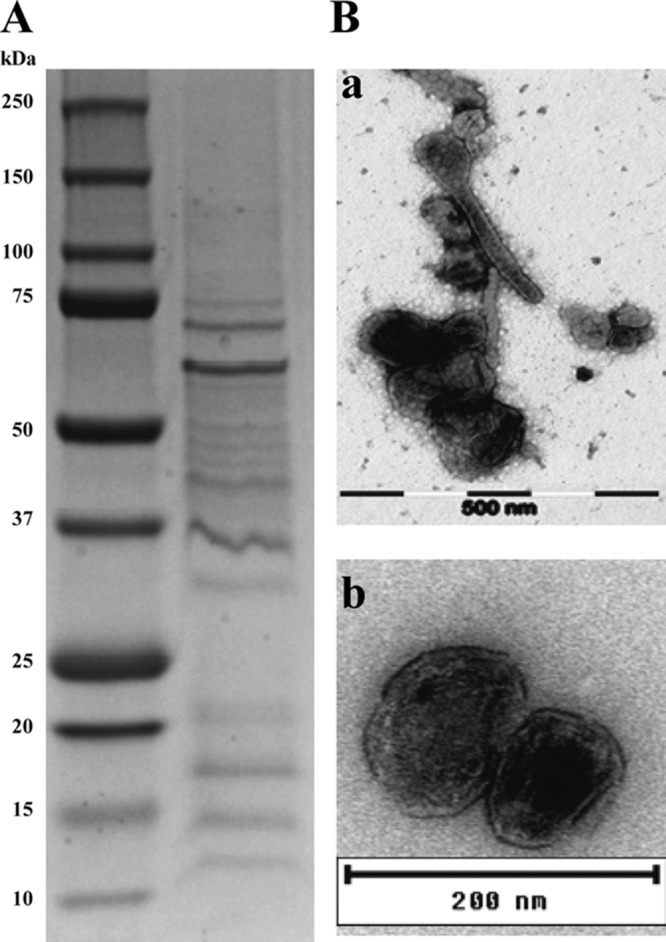
Analysis of MVs isolated from F. noatunensis subsp. orientalis culture supernatant. (A) Analysis of the protein content of MVs by SDS-PAGE and Coomassie blue staining. (B) Electron micrograph of negatively stained MVs isolated from culture supernatant. Size bars indicate 500 nm (panel a) and 200 nm (panel b).

An average of 1 mg of protein, corresponding to 1.6 μg/ml, was recovered from 600 ml of a late-stationary-phase liquid culture of F. noatunensis subsp. orientalis. Several proteins were detected by Coomassie blue staining after separation of the MVs by SDS-PAGE (Fig. 4A). Transmission electron microscopy (TEM) analysis was performed to verify the size, morphology, and integrity of the isolated MVs (Fig. 4B). The MVs were spherical, with a mean diameter of between 60 and 80 nm. In addition, a few nanotubes were observed (Fig. 4B, panel a), which corresponds well to published data for MVs isolated from different species of Francisella (12, 23, 24). Liquid chromatography-tandem mass spectrometry (LC-MS/MS) analysis of three biological replicates from F. noatunensis subsp. orientalis MVs identified 224 proteins (false-discovery rate [FDR], 0.03%) (see Table S2 in the supplemental material). The PSORTb 3.0.2 database identified the potential subcellular localization of 82% of the F. noatunensis subsp. orientalis MV proteins. The majority (∼ 52%) of these proteins were predicted to be cytoplasmic proteins, while 5% corresponded to outer membrane proteins and 1% to extracellular proteins. The identified MV proteins were further categorized on the basis of their predicted functions by using the UniProt database, where proteins involved in translation/transcription, catalytic activity, and transporter activity were the most abundant (18%, 15%, and 15%, respectively) (Table S2). Table 1 lists the top 20 most abundant proteins identified in MVs and their predicted locations. These proteins included homologues of virulence-associated proteins, such as OmpA, GroEL, ClpB, IglC, and IglB, which may contribute to the immunogenic properties of the MVs.

**TABLE 1:** Proteomic characterization of the 20 proteins that are more abundant in OMVs of F. noatunensis subsp. orientalis

Protein	No. of peptide hits	Protein product	Gene locus	Predicted subcellular location
Bifunctional proline dehydrogenase/pyrroline-5-carboxylate	158	WP_014715611.1	OOM_1728	Cytoplasmic
Intracellular growth locus, subunit C (IglC)^*a*^	101	WP_014714932.1	OOM_0933	Unknown
60-kDa chaperonin GroEL^*a*^	92	WP_014714726.1	OOM_0688	Cytoplasmic
ATP synthase subunit b	91	WP_004286980.1	OOM_0555	Cytoplasmic membrane
ATP synthase subunit alpha	89	WP_014714619.1	OOM_0557	Cytoplasmic
Outer membrane-associated protein^*a*^	88	WP_014715233.1	OOM_1284	Outer membrane
Catalase-peroxidase	81	WP_014715162.1	OOM_1206	Unknown
OmpA family protein^*a*^	76	WP_014714420.1	OOM_0329	Outer membrane
Succinate dehydrogenase iron-sulfur subunit	75	WP_014714631.1	OOM_0570	Cytoplasmic membrane
Intracellular growth locus protein B (IglB)^*a*^	73	WP_014714931.1	OOM_0932	Cytoplasmic
Putative uncharacterized protein	63	WP_014714929.1	OOM_0930	Outer membrane
RNase E	59	WP_014715484.1	OOM_1576	Cytoplasmic
Pyruvate dehydrogenase E1 component	58	WP_014714759.1	OOM_0731	Cytoplasmic
Serine-type d-Ala-d-Ala carboxypeptidase	56	WP_014714959.1	OOM_0962	Unknown
Chaperone ClpB^*a*^	53	WP_014715663.1	OOM_1793	Cytoplasmic
Glycerol-3-phosphate dehydrogenase	51	WP_014714672.1	OOM_0621	Unknown
Enolase	51	WP_014715152.1	OOM_1196	Cytoplasmic
Succinate dehydrogenase	48	WP_014714630.1	OOM_0569	Cytoplasmic membrane
Peptidyl-prolyl *cis-trans* isomerase	46	WP_014715675.1	OOM_1806	Outer membrane
ATP synthase gamma chain	45	WP_014714620.1	OOM_0558	Cytoplasmic

a Virulence-associated protein.

### MVs protect adult zebrafish challenged with an acute dose of F. noatunensis subsp. orientalis.

In our previous work, we described the experimental infection of zebrafish larvae with F. noatunensis subsp. orientalis and F. noatunensis subsp. noatunensis. In the present work, we focus on the study of F. noatunensis subsp. orientalis infection in adult zebrafish (with innate and acquired immunity). As shown by others, adult zebrafish are susceptible to infection with F. noatunensis subsp. orientalis by i.p. injection (26). Nevertheless, immersion challenge is a more natural route of infection. Therefore, 35 adult zebrafish were exposed to F. noatunensis subsp. orientalis by static immersion for 1 to 3 h. Fish were exposed to bacterial concentrations of 10^5^, 10^6^, and 10^7^ CFU/ml. Control fish were treated similarly using PBS instead of bacteria. Both control and F. noatunensis subsp. orientalis-exposed fish showed no mortalities or signs of disease over a period of 21 days (data not shown). In the case of E. tarda infection, it has been shown that adult zebrafish are susceptible by both i.p. injection and immersion. However, immersion infection with E. tarda is possible only when it is followed by a dermal abrasion (24). We considered dermal abrasion to be a source of unnecessary stress. Therefore, we conducted the experiment by i.p. injection. Before evaluating the efficacy of MVs isolated from F. noatunensis subsp. orientalis as a vaccine against francisellosis, an F. noatunensis subsp. orientalis-zebrafish dose response experiment was performed (data not shown), obtaining results similar to those reported previously (26). On the basis of these studies, an acute infection dose of 1 × 10^6^ CFU of F. noatunensis subsp. orientalis per fish was chosen (10). Although the use of adjuvant, especially oil adjuvant, is well known in the fish industry, there are reports of side effects, including inflammation, granulomas, and pigmentation at the site of injection and connective tissue in internal organs such as spleen (31). Since the aim of our study was to determine whether MVs can prevent the formation of granulomas caused by subsequent challenge with F. noatunensis subsp. orientalis, the experimental setup did not include extra adjuvants. Moreover, MV-based vaccines have displayed self-adjuvating capabilities in other systems.

**FIG 5:**
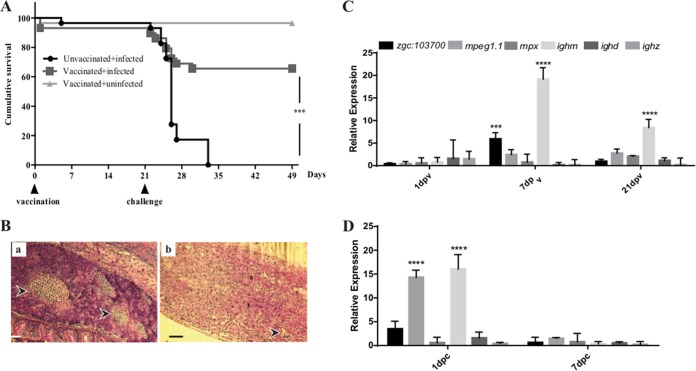
Cumulative survival and immune response of adult zebrafish immunized with MVs and infected with F. noatunensis subsp. orientalis. (A) Kaplan-Meier representation of cumulative survival of adult zebrafish vaccinated with 20 μg MVs and infected with 10^6^ CFU F. noatunensis subsp. orientalis (*P* value, 0.001 [log rank test]). (B) Histological examination by hematoxylin-eosin (HE) staining of spleen from unvaccinated challenged fish 7 dpc (panel a) and MV-vaccinated fish challenged with F. noatunensis subsp. orientalis at 7 dpc (panel b). Arrowheads indicate granulomatous structures. Bars, 150 μm (magnification, ×10). (C and D) Transcriptional response of selected cellular markers of kidney from MV-vaccinated fish before challenge (C) and after challenge (D) as measured by RT-qPCR. Bars represent means ± standard deviations (SD) of relative expression levels compared to those for the control (PBS injected). Relative expression levels were normalized to the expression of *eef1a1l1*. Asterisks indicate significant upregulation (****, *P* < 0.0001; ***, *P* < 0.001).

Of the 65 fish vaccinated i.p. with MVs, 2 died at 1 day postvaccination, due to the handling during vaccination and not related to the vaccine composition. No evidence of discomfort due to vaccination was observed in any other fish. The fish ate and behaved normally, confirming that MVs are safe for immunization. At 21 days after vaccination, both vaccinated and PBS control fish were challenged with F. noatunensis subsp. orientalis (1 × 10^6^ CFU). The vaccinated group exhibited significantly lower mortality than the unvaccinated group (Fig. 5A), in which most deaths occurred between days 2 and 7 after challenge. The unvaccinated group infected with F. noatunensis subsp. orientalis showed signs characteristic of francisellosis such as loss of appetite, lethargy, and reduced swimming. The mortality rate in the unvaccinated group increased rapidly during the first week, where only 20% survived by day 7 after challenge. Vaccinated zebrafish challenged with F. noatunensis subsp. orientalis had a 65.5% survival rate at the end of the experiment (28 days postchallenge [dpc]). Within this group, an initial decrease in appetite was observed during the first 3 days, but this was fully reversed at 4 days postchallenge.

In teleost fish, considering the lack of lymph nodes, spleen and kidney are the main immune organs. These organs presented the greatest number of granulomas, with aggregation of bacteria similar to the response documented by others (26). Histological examination of unvaccinated infected animals at 7 dpc revealed formation of encapsulated granulomas containing small coccoid bacteria in spleen (Fig. 5B, panel a, arrowhead). Averages of 4 to 5 granulomas were observed in spleen, with a diameter from 50 to 200 μm. Some encapsulated granulomas were present in MV-vaccinated fish. The response, however, seemed milder, and the granulomas (between 1 and 2 granulomas with a diameter of 20 to 50 μm per animal) were smaller and more organized (Fig. 5B, panel b, arrowhead). The members of the vaccinated nonchallenged group did not show any sign of lesions in the examined organs.

### Expression profiles of cell markers and cytokines in vaccinated zebrafish.

**FIG 6:**
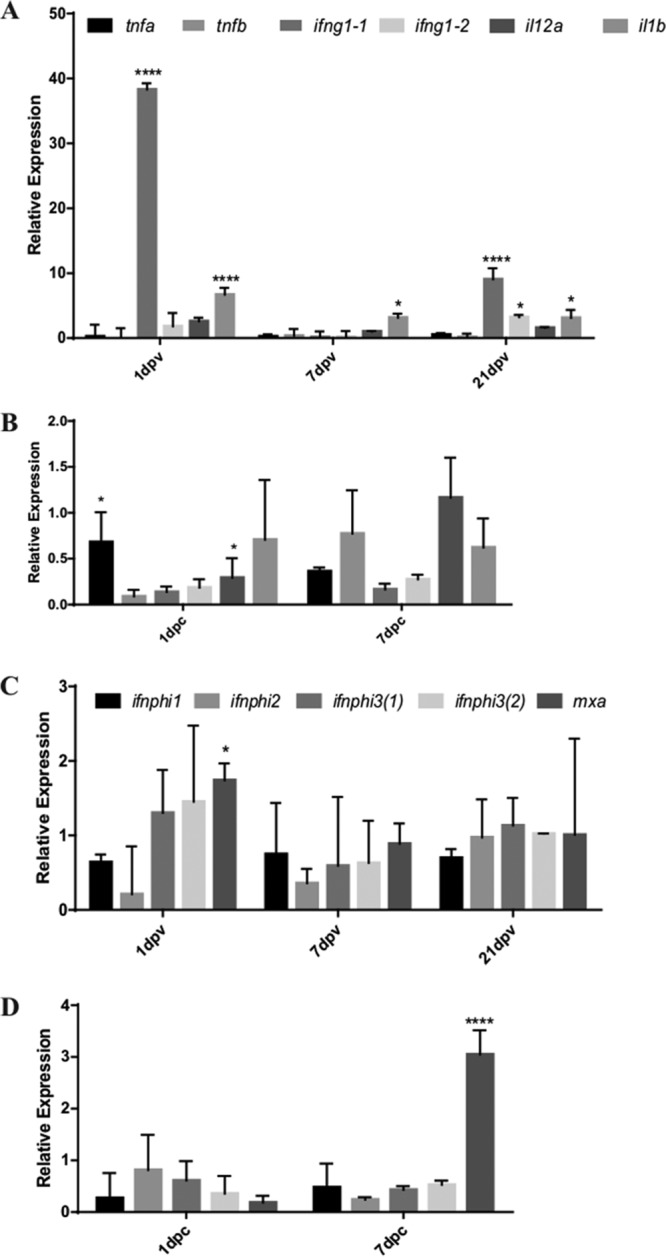
Transcriptional response of cytokine genes in kidney from adult zebrafish. (A and B) Expression patterns of *tnfa*, *tnfb*, *ifng1-1*, *ifng1-2*, *il-12a*, and *il1b* cytokine genes were analyzed after vaccination with MVs (A) and after challenge with F. noatunensis subsp. orientalis (B). (C and D) In addition, cytokine genes involved in the antiviral response, such as *ifnphi1*, *ifnphi2*, *ifnphi3*(*1*), *ifnphi3*(*2*), and *mxa*, were studied after vaccination with MVs (C) and after challenge with F. noatunensis subsp. orientalis (D). Bars represent means ± SD of relative expression levels compared to those for the control (PBS injected). Relative expression levels were normalized to the expression of *eef1a1l1*. Asterisks indicate significant upregulation (****, *P* < 0.0001; *, *P* < 0.1).

The immune response to the vaccination and subsequent challenge was assessed by quantitative real-time PCR (RT-qPCR) at different time points during the experiment. At 7 days postvaccination (dpv), increased transcriptional levels of different cellular markers, such as *zgc*::*103700* (*mhcii*), *mpeg1.1* (macrophage-expressed gene), and *ighm* (immunoglobulin M), were observed. Importantly, at 21 dpv, the most notable increase was in the transcription of *ighm*, suggesting the involvement of B cells in the generation of a protective immune response (Fig. 5C). The participation of mucosal Ig, such as that encoded by *ighd* and *ighz*, was assessed, but neither vaccination nor challenge induced a change in their expression levels (Fig. 5C and D). After challenge, the level of the *mpeg1.1* marker showed a significant increase compared to the PBS-injected control group (Fig. 5D), but no significant upregulation of *mpx* (neutrophil) was observed. These results suggest the participation of macrophages as the main site of infection by F. noatunensis subsp. orientalis, which corroborates the *in vitro* results, where cells of macrophage morphology were the main cell type infected. Differential transcription of the inflammatory cytokines encoded by *il12a*, *il1b*, and *ifng* was observed in the vaccinated group compared to the unvaccinated group before challenge (Fig. 6A). The vaccinated group showed a significantly higher transcriptional level of *ifng1-1*, but not of *ifng1-2*, at 1 dpv, but it had subsided already by 7 dpv and increased again at 21 dpv. The transcription of *il1b* was also increased at 1 dpv compared to the unvaccinated group, with a slight decrease afterwards. In contrast, transcription of *il12a* increased slowly during the postvaccination period; however, even at 21 dpv the increase was not significant. Expression of *tnfa* and *tnfb* was not significant. After subsequent challenge with F. noatunensis subsp. orientalis, no significant expression of inflammatory cytokines was observed (Fig. 6B). Furthermore, in addition to triggering an inflammatory response, MVs might trigger antiviral responses due to their size and the presence of nucleic acid. To investigate this possibility, gene expression analyses of type I *ifnphi1-3*, as well as *mxa*, were included in the panel of the studied genes. In kidney of vaccinated fish, upregulation of *mxa* was observed at 1 dpv (Fig. 6C) and became significant at 7 dpc (Fig. 6D). Only minor upregulation was observed for the type I *ifnphi* genes (Fig. 6C and D).

### Detection of zebrafish immunoglobulin in serum and supernatant by immunoblot analysis.

**FIG 7:**
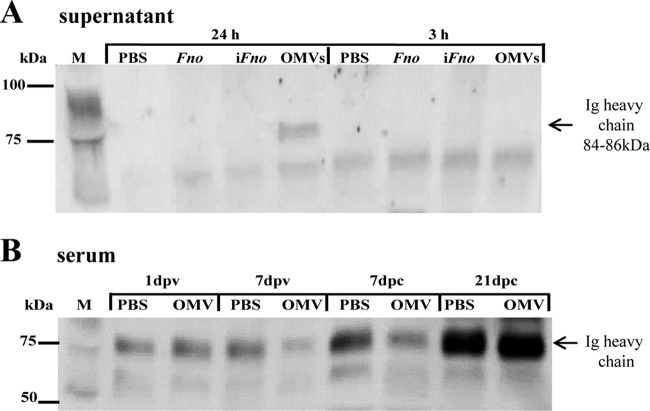
Western blot analysis showing detectable levels of zebrafish IgM. (A) Zebrafish IgM heavy chain (84 to 86 kDa) in supernatant of primary zebrafish kidney cell cultivated *in vitro* for 3 h or 24 h in the presence of PBS, F. noatunensis subsp. orientalis (*Fno*) (1 × 10^8^ CFU/ml), F. noatunensis subsp. orientalis inactivated in formalin (i*Fno*) (1 × 10^8^ CFU/ml), or MVs (20 μg). (B) Zebrafish IgM heavy chain in serum of zebrafish injected with PBS or 20 μg MVs at 1 or 7 days postvaccination (dpv) or at 7 or 21 days postchallenge (dpc) with F. noatunensis subsp. orientalis (1 × 10^6^ CFU). M, marker in kilodaltons (kDa).

In order to investigate the humoral response to F. noatunensis subsp. orientalis, a polyclonal rabbit antibody against the zebrafish Ig heavy chain (kindly donated by Julio Coll) was used to detect total IgM in supernatant from kidney leukocytes and in serum of zebrafish at different time points. Kidney zebrafish leukocytes were cultured *in vitro* in the presence of F. noatunensis subsp. orientalis, i*Fno* (formalin-inactivated F. noatunensis subsp. orientalis), or MVs (20 μg), and the supernatant was collected at 3 h and 24 h. The secretion of IgM was detected only in the sample incubated with MVs for 24 h (Fig. 7A). In pooled sera from zebrafish (*n* = 3), IgM was found to be present in all samples, including those collected both before and after challenge (Fig. 7B). At 21 dpc, protein levels of total IgM were increased in both the unvaccinated and vaccinated groups. It is important to mention that whole IgM was detected and not only the fractions specific for F. noatunensis subsp. orientalis in this assay.

## DISCUSSION

This report is the first to describe the isolation, characterization, and protein identification of MVs from Francisella noatunensis subsp. orientalis. The MVs isolated and characterized in this study contained several proteins capable of mounting a protective response to F. noatunensis subsp. orientalis infection in zebrafish. Importantly, our results demonstrate that MVs induce protection against F. noatunensis subsp. orientalis infection in zebrafish. MVs used as vaccines against other intracellular pathogens have shown good protection in different fish species (16, 20, 21). In zebrafish, the transcriptional immune response to MVs is similar to the response to F. noatunensis subsp. orientalis infection observed in its natural host (tilapia) (5), where F. noatunensis subsp. orientalis induces a potent early inflammatory response marked by the upregulation of *il1b* and *ifng*. Several of the most abundant MV-associated proteins identified through mass spectrometry correspond to genes that were identified previously as part of the proposed Francisella pathogenicity island (FPI) in F. noatunensis subsp. orientalis and its F. tularensis homologues. Many of the FPI-associated virulence factors are essential for the survival of the bacteria inside macrophage and for spread of the disease (7).

In an attempt to understand the pathogenesis of F. noatunensis subsp. orientalis, the main cell types able to internalize these bacteria were investigated using zebrafish as a model. We found that F. noatunensis subsp. orientalis resides within tight vacuoles in the cytoplasm of cells with a morphology characteristic of macrophages in the early phase of infection and that these intracellular bacteria do not seem to be degraded. We also observed that F. noatunensis subsp. orientalis was internalized by granulocytes, where the bacteria were already degraded within 1 h after the infection. Thus, we hypothesize kidney macrophages to be the main target for francisellosis infection. The presence of bacteria inside tightly fitting vacuoles is consistent with previous reports on the morphology of attenuated human vaccine strain F. tularensis LVS within human and mouse macrophages (18). For F. tularensis, the current understanding is that a bacterium is initially localized inside phagosome and subsequently colocalized with late endosomal marker protein 1 (LAMP-1). Electron microscopy studies indicate that F. tularensis escapes from late endosome/phagosome within a few hours after uptake and appears to be free in the cytoplasm or within organelles with incomplete membranes (18, 30). Similar electron microscopy observations of cytosolic bacteria were made in F. noatunensis subsp. noatunensis-infected Dictyostelium discoideum amoebas (25). Further studies are needed to explore the ability of F. noatunensis subsp. orientalis to replicate and escape from the phagosome in macrophages, as described for other species of Francisella.

Bacteria, especially those that cause chronic infections in their host organisms, have developed a wide range of strategies to evade the innate and adaptive immune response, for example, release of MVs, which helps establish the infection (8–10, 17, 19). Even though the secretion of MVs is a common feature of several bacterial species, including F. noatunensis subsp. noatunensis and F. tularensis subsp. novicida, the ability of F. noatunensis subsp. orientalis to secrete MVs had not been previously investigated. For this study, isolation and characterization of MVs from F. noatunensis subsp. orientalis are reported. The secretion of MVs was observed at stationary-growth phase at an optical density at 600 nm (OD_600_) of 5 to 6, indicating that certain stress factors, such as lack of nutrients, may increase the release of MVs. It has been reported that differences in the protein content of MV are dependent on strain and growth conditions (32). LC-MS/MS analysis of F. noatunensis subsp. orientalis-derived MVs identified 224 proteins. Approximately 52%, including chaperon GroEL and heat shock protein ClpB, were predicted to be cytoplasmic proteins. GroEL is a chaperon protein that has been suggested to induce the expression and release of interleukin-6 (IL-6) and tumor necrosis factor alpha (TNF-α) in human monocytes (33), while ClpB is a heat shock protein with a strong immunogenic effect (34). The identification of catalytic proteins in MVs has been reported in several species (9, 35), and packing of active enzymes into MVs has been proposed to have an important role in virulence. Thus, the presence of enzymatic proteins within MVs may provide increased bacterial survival and resistance during infections (12). Interestingly, all of the identified *Fno*-MV proteins have previously been identified as components of MV derived from other Francisella species (12, 15).

Some of the most interesting genes identified in F. tularensis belong to the intracellular growth loci (*iglA*, *iglB*, *iglC*, and *iglD*) which are part of the Francisella pathogenicity island (FPI). Expression of these genes is essential for the survival and growth of the bacteria inside macrophages (36). *iglC* mutants are not able to grow inside macrophages, are deficient in the ability to escape from phagosome, and fail to downregulate the proinflammatory response (37). Homologues of the F. tularensis subsp. *novicida iglA*, *iglB*, *iglC*, and *iglD* genes have previously been identified in F. noatunensis subsp. orientalis and F. noatunensis subsp. noatunensis (7, 16). *iglC-Fno* mutants have been tested as a vaccine against francisellosis in tilapia, with relative survival rates of 68.75% to 87.5% (7). Thus, it can be argued that the high abundance of GroEL, ClpB, IglC, and IglB proteins in F. noatunensis subsp. orientalis-derived MVs might contribute to an increased immunogenic effect of MVs.

In mammals, gamma interferon (IFN-γ) seems to be crucial in the control of F. tularensis infection (38). In a mouse macrophage model of Listeria monocytogenes infection, IFN-γ activation prevented escape of the bacterium from phagosomes and led to subsequent killing of the bacterium (39). In the present study, *Fno*-MVs in zebrafish induced a significant upregulation of *ifng1-1* transcription at 24 h postvaccination and the upregulation was maintained until 21 days postvaccination. Expression of *ifng1-2* showed no significant changes. The presence of two different *ifng* genes (*ifng1-1* and *ifng1-2*) seems to be the rule in teleosts, and they may have distinct expression ranges and functions depending on the fish species (40). Recent *in vivo* studies of IFN-γ target gene induction showed that the responses elicited by *ifng1-1* and *ifng1-2* were identical for the two *ifng* genes (41). Morpholino-mediated knockdown of *ifng* genes does not render the embryos susceptible to bacterial infection. However, *in vitro* cross-linking experiments performed with recombinant extracellular domains of potential goldfish receptors have shown that *ifng1-1* and *ifng1-2* do not use the same receptor complexes (41, 42). The emerging picture of the *ifng* system appears more complex in fish than in mammals. This complexity includes several ligands and receptors for the same molecules, where the response depends on the type of cells and the receptor complexes that they express. TNF-α has been shown to play a central and complex role in the resolution of F. tularensis Schu4 infection (43). In studies using a low multiplicity of infection in peripheral blood mononuclear cells, a slight delay was observed in the induction of TNF-α expression compared to the inflammatory response (43). The existence of two TNF-α genes has been reported for several fish species, including zebrafish (44). A probable reason for the existence of multiple isoforms of cytokines is the occurrence of genome duplication in bony fish. It has been suggested that the zebrafish TNF-α gene is the acute- and longer-response type in zebrafish and that the TNF-β gene is the delayed-response type. Here we observed upregulation of the TNF-α gene at 1 dpc, suggesting that MVs could induce a longer acute response. Collectively, the data suggest that MVs might mimic the infection of F. noatunensis subsp. orientalis but at a low infection dose. Cytokine IL-12 is composed of two different subunits, IL-12p35 and IL-12p40, where the p40 subunit is shared with other cytokines, for example, IL-23. In the present study, a significant increase in the expression of *il12p35* (*il12a*) was not detected, suggesting that IL-12 was not upregulated. In future experiments, it might be interesting to evaluate the expression of the other subunit (*p40*) which, together with il23p19, forms the functional IL-23. This cytokine plays an important role in the development of the Th17 subset and in innate immune responses (45). Recent studies indicated the presence of small RNAs (sRNAs) in MVs, showing that bacterial RNA can be released from bacteria via membrane vesicles. Koeppen et al. demonstrated that MVs produced by an extracellular pathogen (Pseudomonas aeruginosa) deliver sRNAs to host cells. This is a novel mechanism of pathogen-host communication that regulates the immune response in human airway epithelial cells and in mouse lung (46). Considering these characteristics of MVs, the expression patterns of antiviral genes, such as *ifnphi1-3* and *mxa*, were also analyzed in this study. The expression and antiviral activity of IFNs and Mxa have been extensively described in zebrafish. Viral hemorrhagic septicemia virus (VHSV)-infected zebrafish showed an increase in *ifna*, *ifnb*, and *mxa* gene expression, reaching a maximum after 72 h (47). In our case, we did not detect significant changes in the expression of *ifnphi1-3* genes. However, we observed a significant increase in expression of *mxa* at 1 dpv and especially at 7 dpc. This suggests the possibility that MVs could be acting as a delivery system of different bacterial components, including RNA and DNA. Further experiments are needed to elucidate the characteristics and quality of the genetic material packed inside MVs. In investigations of the expression of different cellular markers, MVs induced the upregulation of *mpeg1.1* following infection with F. noatunensis subsp. orientalis at 1 dpc, indicating activation of macrophages. The macrophage-expressed gene (*mpeg1.1*) was first identified as a gene with expression tightly restricted to human and murine macrophages and has subsequently been used as a marker for this cell lineage in mammals and zebrafish (28). Recent studies using the zebrafish-M. marinum infection model have shown that *mpeg1.1*-expressing macrophages participate in the formation of a granuloma-like structure and that knockdown of *mpeg1.1* results in an increased bacterial burden. This is consistent with the proposed bacterial function of *mpeg1.1* as a pore-forming molecule (48). The upregulation of *mpeg1.1* after F. noatunensis subsp. orientalis infection confirms our results obtained *in vitro* where cells with macrophage morphology were identified as the main cell type infected with F. noatunensis subsp. orientalis. To date, three major Ig isotypes have been reported in teleost fish, where IgM was the first discovered decades ago, while IgD and IgT/IgZ were discovered later. Little is currently known about the IgT-positive (IgT^+^)/IgZ^+^ and IgD^+^ B cell populations secreting these Ig classes in the mucosal sites. Therefore, the antibody availability has until now been restricted to IgM. The delivery method of one given pathogen or immunogen is a determinant of Ig responses, where serum IgM production is often much higher when antigens are systemically injected than when they are delivered by any of the mucosal routes. IgM is generally composed of either two heavy chains (82 and 50 kDa) in the case of carp and tilapia or one heavy chain (84 to 86 kDa) and one light chain (20 to 25 kDa) (49) in zebrafish. In the present study, vaccination with MVs induced upregulation of *ighm* at 7 and 21 dpv. Furthermore, the upregulation of *ighm* expression was maintained for up to 1 dpc, suggesting activation of B cells. Besides, the heavy chain of IgM was detected in the supernatant of zebrafish leukocytes cultured *in vitro* in the presence of MVs for 24 h. After 3 weeks following the vaccination of fish with MVs, a relatively weak antibody response was observed that was not significantly different from the one observed in the control group. However, by 21 days postchallenge with F. noatunensis subsp. orientalis, an increase in the antibody response was detected in the serum of vaccinated as well as unvaccinated fish, suggesting the protective role of the humoral response during F. noatunensis subsp. orientalis infection. It would be interesting to see whether there are quantitative differences between unvaccinated and vaccinated fish in the strength/titer or in the diversity of humoral responses specific for F. noatunensis subsp. orientalis antigens. However, the small amount of serum obtainable from small fish such as zebrafish precludes such assays. Studies in zebrafish infected with rhabdovirus, such as viral hemorrhagic septicemia virus (VHSV), have shown that plasma from surviving zebrafish has a neutralizing activity in fish cells *in vitro* and that the neutralization correlated with the level of *in vivo* protection (47). Synergy between antibodies, cytokines, and phagocytes against intracellular pathogen appears to be critical for clearance of an infection. In many Gram-negative bacteria, the protective antibody may act in conjunction with the complement system, which enhances the ability of phagocytic cells to kill pathogens. The importance of an antibody-mediated response has been studied previously for F. noatunensis subsp. orientalis, with results showing that a specific antibody response is a useful component of the protective immune response to F. noatunensis subsp. orientalis in tilapia (7). In our study, we observed activation of all those components during F. noatunensis subsp. orientalis infections, although the exact mechanism has yet to be determined.

The use of MVs as vaccines has been investigated in several other systems, including mice, humans, and fish. They are particularly attractive as vaccines due to the protective capabilities against intracellular bacterial infections, which represent a challenge in aquaculture (1–3). They are also desirable for their small size, which makes them act as nanoparticles, and for their ability to deliver a variety of immunogenic factors. The versatility allows diverse routes of administration, including i.p. injection, feed or pellet coating (oral administration), and bath vaccination. Further experiments are needed to determine the best route of immunization and perhaps whether it is possible to combine MVs with other vaccines or adjuvants.

In summary, F. noatunensis subsp. orientalis infects mainly cells with macrophage morphology and is able to secrete MVs that contain known virulence factors. Vaccinations with F. noatunensis subsp. orientalis MVs induce protective immunity against francisellosis in a zebrafish infection model. Further studies are required to investigate whether this also applies for tilapia, the natural host of F. noatunensis subsp. orientalis. Nevertheless, MV vaccination represents a promising candidate to protect different fish species against intracellular pathogens.

## MATERIALS AND METHODS

### Strains, media, and labeling.

Cultivation of F. noatunensis subsp. orientalis (07-285A; isolated from diseased tilapia Oreochromis niloticus in Costa Rica) (5) was performed using Eugon chocolate agar (ECA) or Eugon broth supplemented with 2 mM FeCl_3_ (EBF) as previously described (27) with agitation (100 rpm) at 20°C. Bacterial stocks were frozen in autoclaved 10% skimmed milk (BD Difco) or in BD Bacto Eugon broth supplemented with 20% glycerol (Sigma-Aldrich) and stored at −80°C. The number of CFU for each experiment was estimated by plating on an ECA plate 10 μl of 10-fold serial dilutions of a bacterial suspension. Labeling with FITC was performed following the procedure of Hazenbos et al. (50). Briefly, bacteria from overnight cultures were pelleted, resuspended in PBS, and adjusted to an OD_600_ of 2 (∼3.5 × 10^9^ CFU/ml). Bacteria (3.5 × 10^8^) were transferred to a microcentrifuge tube, pelleted, and suspended in 1 ml of 0.5 mg/ml FITC (Sigma)–50 mM sodium carbonate–100 mM sodium chloride solution. Bacteria were incubated for 20 min at room temperature, washed three times in 1 ml of HBSA (Hanks' buffer supplemented with 0.25% bovine serum albumin and 20 mM HEPES buffer) at 34,500 × *g* and 4°C for 10 min, and then resuspended in 100 μl of PBS.

### Isolation of MVs.

A 10-ml volume of an overnight liquid culture of F. noatunensis subsp. orientalis was used to inoculate 200 ml EBF to reach an OD_600_ of approximately 1, and bacteria were then grown to the late stationary phase. Bacteria were pelleted at 15,000 × *g* and 4°C for 10 min. The supernatant was sequentially filtered through 0.45-μm- and 0.2-μm-pore-size filters. The filtrate was then subjected to ultracentrifugation in a Sorvall Discovery 100 Ultracentrifuge (Sorvall) at 100,000 × *g* and 4°C for 2 h and the supernatant discarded. The pellet was washed with 50 mM cold HEPES buffer (pH 6.8) and subjected to ultracentrifugation for an additional 30 min at 100,000 × *g* and 4°C to pellet the MVs. Finally, the supernatant was discarded and the MVs were resuspended in 100 μl PBS (pH 7.2). Protein concentration was determined by the use of a NanoDrop spectrophotometer (UV5 nano; Mettler Toledo). MV aliquots (100 μl) were plated onto ECA plates and incubated at 20°C for at least 3 weeks to ensure sterility. The remaining sample was stored at −80°C.

### Francisella infections of adult zebrafish.

Male and female zebrafish (Danio rerio) of the wild-type AB strain or the transgenic *Tg*(*mpx:GFP*)^*i114*^ strain were obtained from the Model Fish Unit at the Norwegian University of Life Sciences. Fish were 10 to 11 months old and kept in 6-liter fish tanks. They were fed every morning with brine shrimp (Scanbur AS, Nittedal, Norway) and every afternoon with SDS 400 Scientific Fish Food (Scanbur AS). Fifty percent of the water was changed daily. The following water parameters were monitored every third day using commercial test kits (TetraTest kit; WebZoo AS, Heggedal, Norway): pH, NO_2_^−^, NO_3_^2−^, NH_3_/NH_4_^+^, and water hardness. Experimental fish were anesthetized by immersion in water containing 100 mg/ml tricaine methanesulfonate (MS-222; Sigma-Aldrich), placed on a wet sponge, and injected using a 27-gauge needle into the midline of the coelomic cavity, posterior to the pectoral fins. Fish were injected with a total of 15 μl of suspension. Four fish were injected per needle. After injection, the fish were returned to recovery tanks immediately and kept in separate 10-liter tanks. Infected and control fish were held in static 15-liter tanks with lids, in groups of 35 fish per tank. The tanks were housed in a water system with a controlled temperature (27°C) and with a 14-h/10-h light/dark cycle. The fish were closely monitored, and mortality was recorded twice a day. For the vaccine experiment, 65 fish per group were immunized once with 20 μg MVs (vaccinated group) or PBS (unvaccinated group) by i.p. injection. An extra group of 65 fish was also immunized once with 20 μg MVs and used as a vaccine control group (vaccinated nonchallenged group). After 21 days (600 degree-days), the fish were challenged by i.p. injection with a challenge dose of 1 × 10^6^ CFU F. noatunensis subsp. orientalis (infected group) or PBS (vaccinated uninfected group). The challenge dose selected for the vaccine experiment was chosen according to our dose-response results and as described in the literature (26, 27). For the study of F. noatunensis subsp. orientalis infection *in vivo*, wild-type or transgenic zebrafish expressing neutrophil-specific GFP reporter *Tg*(*mpx:GFP*)^*i114*^, obtained from S. A. Renshaw's laboratory (51), were injected with PBS or 1 × 10^6^ CFU F. noatunensis subsp. orientalis transformed with the mCherry-expressing plasmid pKK289Km/*mCherry* (*Fno*-mCherry), as described by Brudal et al. (27). Flow cytometry was performed at 2, 24, and 48 h after infection. All zebrafish experiments were approved by NARA (the Norwegian Animal Research Authority), and wastewater was decontaminated by chlorination and tested for sterility before disposal.

### Flow cytometry and sorting.

Zebrafish were anesthetized in tricaine methanesulfonate (MS-222; Sigma-Aldrich). Zebrafish were bled following the standardized protocol published by Babaei et al. (52), and organ recollection was performed as described by others (28). For *in vitro* experiments, 10 whole kidneys were pooled in 1 ml medium (Leibovitz L-15 medium [Gibco] supplemented with 2% fetal bovine serum [FBS], penicillin [10 U/ml], and streptomycin [10 μg/ml]). For *in vivo* experiments, single organs were analyzed individually. Single-cell suspensions were generated by gentle teasing of the tissue on a 40-μm-pore-size cell strainer with a plunger from a 1-ml syringe, collected in a 50-ml tube, and rinsed twice with 1 ml of medium. The cells were cultivated at a concentration of 1 × 10^6^ cells/ml in a 24-well plate. The ability of kidney cells to take up F. noatunensis subsp. orientalis *in vitro* was measured following methods described previously (53). Briefly, 1 ml of isolated kidney leukocytes (1 × 10^6^ cells) per sample was incubated for 1 h at 20°C with *Fno*-FITC (1 × 10^8^ CFU/ml), inactivated *Fno*-FITC (1 × 10^8^ CFU/ml), or PBS as a control, without any centrifugation step to enhance the infection. The experiment was performed at 20°C to simulate the optimal temperature for harvest of bacterial cultures and outer membrane vesicles (OMV). After the incubation, cells were centrifuged at 600 × *g* for 10 min and the supernatant was collected and stored at −20°C until analysis by Western blotting. The pellet was washed three times with ice-cold PBS and analyzed by flow cytometry using a Beckman Coulter Gallios flow cytometer. At least 10,000 events were collected for each sample. Data were analyzed using Kaluza software v.1.2 (Beckman Coulter) and lymphocytes gated using side scatter (SSC) (granularity) and forward scatter (FSC) (size) parameters. Discrimination of aggregates from singlets was performed using side scatter-W (SSC-W) versus side scatter (SSC), and Hoechst stain was combined with propidium iodide stain for the separation of dead and live cells (54). The fluorescence of FITC-conjugated F. noatunensis subsp. orientalis was measured before and after the addition of trypan blue (0.025% final concentration) to quench extracellular fluorescence. Incorporation of *Fno*-FITC was measured at 520 nm (FL1). The cell sorting was performed following the same setup using BD FACSDiva software in a BD FACSAria II cell sorter (BD Bioscience) in the Flow Cytometry Core Facility at the Oslo University Hospital.

### Transmission electron microscopy (TEM).

MVs were subjected to negative staining for TEM analysis. Formvar- and carbon-coated copper grids were incubated on a drop of an MV suspension for 5 min. The grids were then washed three times with PBS and the adhered MVs fixed in 1% glutaraldehyde (Sigma-Aldrich) for 4 min. Next, grids were washed three times with PBS and two times with Milli-Q (MQ) water, stained for 20 s with 4% uranyl acetate (Sigma-Aldrich)–MQ water, washed once with MQ water, and finally incubated on a solution of 1.8% methyl-cellulose (Sigma-Aldrich) and 0.4% uranyl acetate for 10 min on ice. The grids were then dried and viewed in a Philips CM200 transmission electron microscope. Images were acquired using a Quemesa camera and iTEM software (both soft imaging solutions [Olympus, Germany]). For ultrastructural analysis of infected kidney cells after cell sorting, 1 × 10^6^ cells were fixed with 1% glutaraldehyde–0.2 M HEPES buffer (pH 7.4) overnight. Cells were washed and embedded in 1% low-melting-point agarose (Thermo Fisher Scientific, MA, USA), postfixed with 2% osmium tetroxide (Electron Microscopy Sciences, EMS, PA, USA), and subjected to contrast staining performed with 2% uranyl acetate (EMS), both for 1 h. Dehydration was performed with a graded ethanol series (70% to 80% to 90% to 95% to 100%), followed by progressive infiltration with epoxy resin (Sigma) over 2 days and polymerization overnight at 60°C. 70-nm-thick sections were cut using an Ultracut UCT EM ultramicrotome (Leica Microsystems, Austria) and a diamond knife (Diatome, Switzerland) and were subjected to contrast staining performed with 0.2% lead citrate for 15 s. Samples were analyzed with a JEM-1400 transmission electron microscope (JEOL). Images were taken with a TemCam-F216 camera and EM-MENU software (TVIPS, Germany).

### In-solution digestion and protein sequence analysis by LC-MS/MS.

Three biological replicates of F. noatunensis subsp. orientalis-derived MVs that had been stored at −80°C were thawed and diluted to 40 μg of total protein in PBS, and the pH was adjusted to 8 by adding ammonium bicarbonate (Sigma-Aldrich). The samples were then subjected to in-solution digestion by adding 5 μl of reduction solution (200 mM dithiothreitol [DTT] [Sigma-Aldrich] dissolved in 50 mM ammonium bicarbonate) to the samples followed by incubation for 60 min at 37°C. After incubation, 10 μl of 200 mM iodoacetamide (Sigma-Aldrich) dissolved in 50 mM ammonium bicarbonate was added to the samples and they were incubated in the dark at room temperature for 60 min followed by trypsin digestion at a 1:20 ratio (0.1 μg/μl trypsin [Promega, sequencing grade] dissolved in 50 mM ammonium bicarbonate) and then incubated overnight at 37°C. After digestion, 10 μl of 5% formic acid (Sigma-Aldrich) was added, the samples were dried in a vacuum centrifuge, and the peptides were reconstituted in 0.2% (vol/vol) formic acid and 2% (vol/vol) acetonitrile (Sigma-Aldrich). The peptides were purified using ZipTip C_18_ (Millipore) according to the manufacturer's instructions prior to mass spectrometry. Trypsin-digested peptides were analyzed as routinely performed by the Australian Proteome Analysis Facility (APAF), using nanoflow liquid chromatography-tandem mass spectrometry performed on a VelosPro linear ion trap mass spectrometer (Thermo Scientific).

### Proteomic data analysis.

Raw data files were converted into mgf format and processed through global proteome machine (GPM) software using version 2.2.1 of X!Tandem, and a nonredundant output file was generated for protein identifications with log (E) values of less than −1. Peptide identification was determined using a fragment ion tolerance value of 0.8 Da. MS/MS spectra were searched against the Francisella noatunensis subsp. orientalis (strain Toba 04) genome (55), and reverse database searches were used in the estimation of false-discovery rates. The three protein identification output files from each sample were combined to produce a single merged output file for the fractions of MVs. Restricting the analysis to proteins reproducibly in all three replicates, and further requiring a total spectrum count of at least six, resulted in the minimum number of peptides used to identify each protein, with an average of two per replicate. The subcellular location and functions for each of the identified MV proteins were predicted using PSORTb 3.0.2 and The UniProt database.

### RNA isolation and quantitative real-time PCR.

Zebrafish tissues were sampled at 1, 7, and 14 days after vaccination and at 1 and 7 days after challenge. Each sampling point used 5 fish per group. Kidneys were recollected individually from euthanized fish. The organs were kept in RNAlater (Ambion) and stored at 4°C until further processing. The tissue was homogenized in 600 μl with buffer RLT (supplemented in an RNeasy minikit; Qiagen) using a mortar and pestle (Sigma-Aldrich), followed by passage of the lysate through a blunt 20-gauge needle fitted to a small, 1-ml syringe (BD). Total RNA was extracted using a Qiagen RNeasy kit, including a 15-min on-column DNase treatment using an RNase-free DNase set (Qiagen), according to the instructions of the manufacturer. The RNA was diluted in 30 μl RNase-free H_2_O (Qiagen). RNA quantity and quality were measured with a NanoDrop spectrophotometer (UV5nano; Mettler Toledo). Reverse transcription reactions were performed by using a High Capacity RNA-to-cDNA kit (Applied Biosystems). Quantitative real-time PCR (RT-qPCR) was carried out for each of the sampling points for a defined set of genes. QuantiTec bioinformatically validated primers were obtained from Qiagen for *il-12p35*, *ifn-γ1-1*, *ifn-γ1-2*, *ifnphi1*, *ifnphi2*, *ifnphi3*(*1*), *ifnphi3*(*2*), and *mx-a*. The remaining primers were obtained from Life Technologies Inc. Information on the primers is listed in Table S1 in the supplemental material. RT-qPCR was performed in triplicate using a LightCycler 480 instrument (Roche) as previously described by Brudal et al. (27). 18S and elongation factor-1 alpha (*eef1a1l1*) genes were used as the reference genes for normalization of the relative transcription levels of each gene, and the normalized immune response data of OMV-injected fish were standardized against the transcription levels of PBS-injected fish for each time point. Relative expression levels were calculated using the Pfaffl method (56), with efficacy correction performed for each primer.

### Histologic sample preparation.

Whole prefixed zebrafish went through an ethanol series of 70%, 80%, 90%, 95%, and 3× 100% ethanol, with each step lasting 1 to 2 h at room temperature. Ethanol was replaced by a preparation solution (Technovit 7100 with hardener I; Heraeus Kulzer GmbH, Wehrheim, Germany) according to the manufacturer's protocol, and the reaction mixture was incubated on a rotating table at room temperature for 2 days. Fish were transferred to separate silicone molds, and 50 μl of hardener II ml^−1^ of the preparation solution was mixed and added to fill the molds. The resin was allowed to harden at room temperature for 1 to 2 h before samples were incubated overnight at 37°C. Resin-embedded fish were mounted on Histobloc (Heraeus Kulzer) holders with Technovit Universal Liquid mixed with Technovit 3040 according to the manufacturer's protocol. Sections (3 μm in thickness) were prepared using a Leica RM2245 microtome and TC-65 Leica tungsten carbide disposable blades (Leica Biosystems Nussloch, Germany), transferred to a water bath, and positioned on glass slides. The sections were dried at 50°C on an HP-3 Kunz Instruments heating plate for 10 to 15 min before staining with hematoxylin and eosin was performed.

### SDS gel electrophoresis and Western blot analysis.

For SDS-PAGE procedures, 20 μg of MVs isolated from F. noatunensis subsp. orientalis was loaded onto a 12% (wt/vol) SDS polyacrylamide gel. The proteins separated by SDS-PAGE were stained with Coomassie blue, and an image was acquired and evaluated using Gel doc XR+ with Image Lab software (Bio-Rad). Immunoblot analysis was used to detect the presence of the heavy chain of zebrafish IgM in blood plasma or in supernatant of zebrafish kidney cells grown *in vitro*. To perform immunoblotting, ∼3 μg of zebrafish plasma protein per lane or 25 μl of supernatant was loaded on a 4% to 15% Mini-Protean gel (Bio-Rad) and then transferred to a nitrocellulose membrane. In order to standardize the amount of protein loaded on a gel, the protein concentration in serum was determined using a NanoDrop spectrophotometer and the membrane was stained with Ponceau Red (2% Ponceau Red, 6% acetic acid) followed by washing in distilled water (see Fig. S3 in the supplemental material). A membrane was blocked with 5% skim milk before incubation for 1 h at room temperature with rabbit anti-CH4 zebrafish IgM (a kind gift by Julio Coll). After washing with PBS–0.05% Tween 20 (PBS-T) was performed, a membrane was incubated with 5,000-fold-diluted anti-rabbit horseradish peroxidase (HRP)-conjugated IgG (Santa Cruz). Finally, bands were visualized by chemiluminescence with a Luminata Crescendo Western HRP substrate (Millipore) in a Chemi Genius Bio-imaging system (Syngene).

### Statistical analysis.

Data (means ± standard deviations [SD]) were analyzed (Prism 6.0; GraphPad Software Inc.) using unpaired, two-tailed *t* tests for comparisons of results from 2 groups and one-way analysis of variance (ANOVA) with Turkey's multiple-comparison method (*, *P* < 0.03; **, *P* < 0.001; ***, *P* < 0.001; ****, *P* < 0.0001). Kaplan-Meier survival curves were used to analyze percentages of survival, and the statistical significance of differences between groups is indicated by three asterisks (***, *P* < 0.001 [Gehan-Breslow-Wilcoxon test and log rank test]).

### Ethics statement.

All animal experiments were approved by the Norwegian Animal Research Authority (approval no. 15/192493-1, FOTS ID 8053), and animals were treated according to institutional guidelines.

## Supplementary Material

Supplemental material
