# Supplementary material for: Characterization and Vaccine Potential of Membrane Vesicles Produced by Francisella noatunensis subsp. orientalis in an Adult Zebrafish Model
Source: Clin Vaccine Immunol. 2017 May 5;24(5):e00557-16. doi: 10.1128/CVI.00557-16 (PMC5424235; doi:10.1128/CVI.00557-16)
Supplement: Supplemental material [file supp_24_5_e00557-16__index.html]

Characterization and Vaccine Potential of Membrane Vesicles Produced by Francisella noatunensis subsp. orientalis in an Adult Zebrafish Model — Supplemental material 

# Characterization and Vaccine Potential of Membrane Vesicles Produced by Francisella noatunensis subsp. orientalis in an Adult Zebrafish Model

## Supplemental material

- Supplemental file 1 -

  Fig. S1. Gating strategy of the major cell lineages from kidney by light-scatter characteristics. Fig. S2. Incorporation of *Fno*-mCherry *in vivo* analyzed by flow cytometry. Fig. S3. Representative Western blot membrane showing protein content. Table S1. Primers used in qRT-PCR analysis. Table S2. Proteins Identified in OMVs from *Francisella noatunensis* subsp. *orientalis*. Table S3. Threshold cycle values from the RT-qPCR data presented in Fig. 6.

  PDF, 6.3M
