# Supplementary material for: Characterization and Vaccine Potential of Membrane Vesicles Produced by Francisella noatunensis subsp. orientalis in an Adult Zebrafish Model
Source: Clin Vaccine Immunol. 2017 May 5;24(5):e00557-16. doi: 10.1128/CVI.00557-16 (PMC5424235; doi:10.1128/CVI.00557-16)
Supplement: Supplemental material [file CVI.00557-16_zcd999095474s1.pdf]

Figure 1S

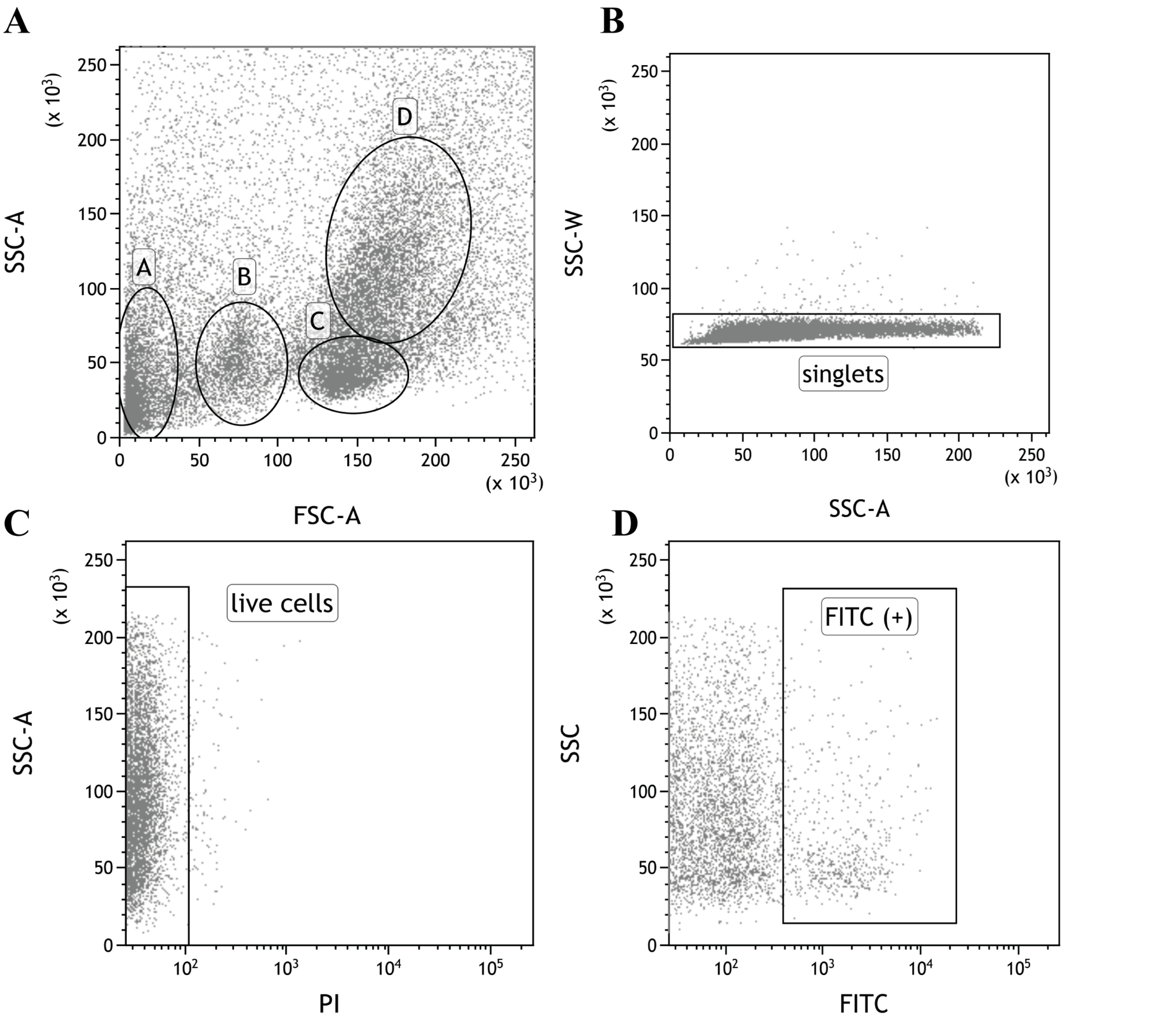

Figure 1S. Gating strategy of the major cell linages from kidney by light-scatter characteristics. **A.** The different gates shown the different cell subset using forward scatter (FSC) and side scatter (SSC). A: mature erythrocytes, B: lymphocytes, C: precursors, D: mielomonocyt cells. **B.** Discrimination of aggregates from singlets using SSC-W versus SSC. **C.** Cell viability using propidium iodine. **D.** Cells able to incorporate Fno-FITC. Representative experiement of three idenpendent experiments.

Figure 2S

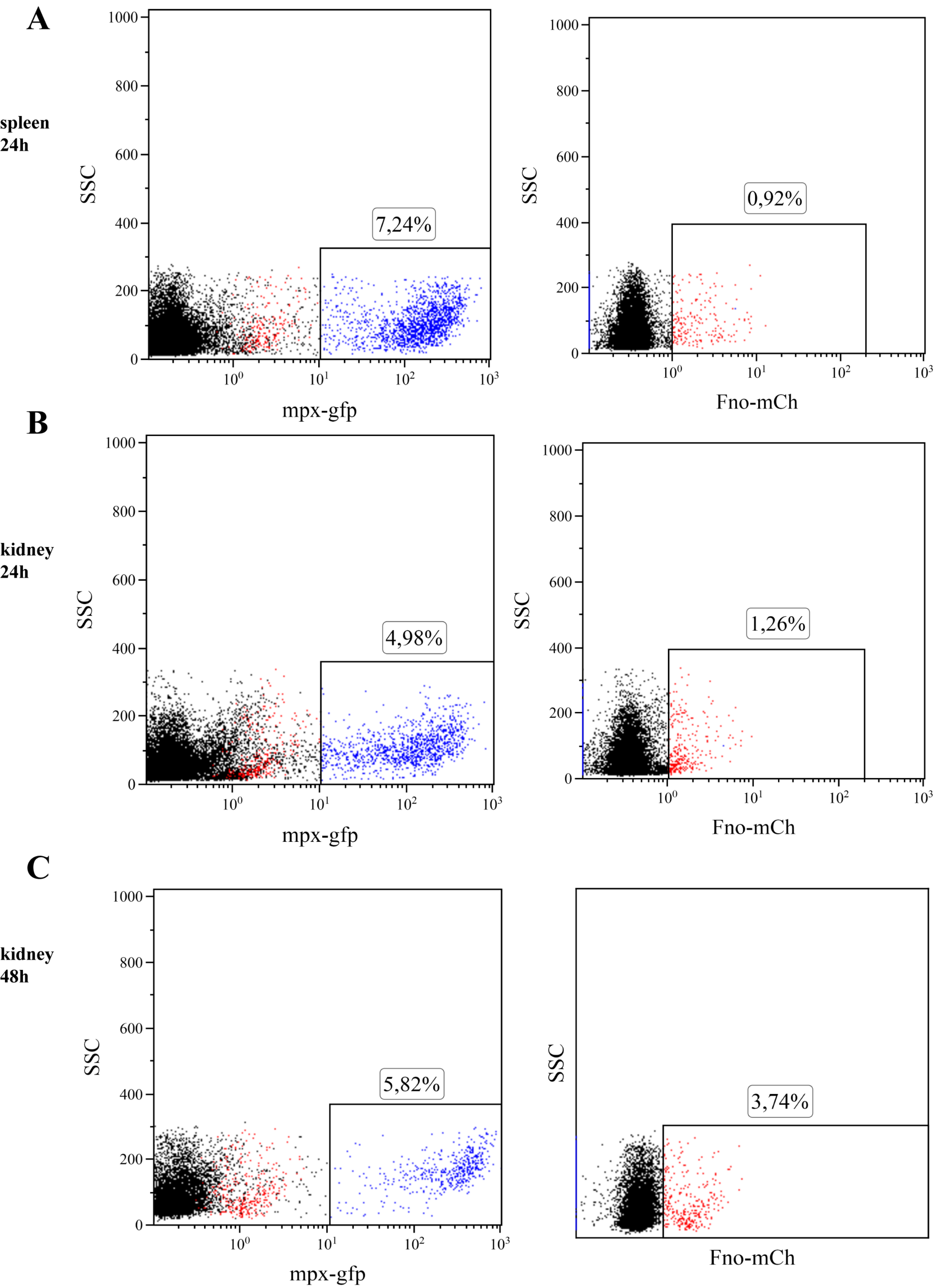

Figure 2S. Incorporation of *Fno*-mCherry *in vivo* analyzed by flow cytometry. Transgenic adult zebrafish *mpx*-gfp were challenge with *Fno*-mCherry and the incorporation of *Fno* was analyzed at different time points. Spleen (A) and kidney (B) were isolated at 24 h or 48 h (C) after injection and analyzed by flow cytometry. Dot-plot show the percentage of neutrophil (*mpx*-gfp) (left column) and the percentage of cells able to incorporate *Fno*-mCherry (right column). The data are based on representative experiment of three replicates.

Figure 3S.

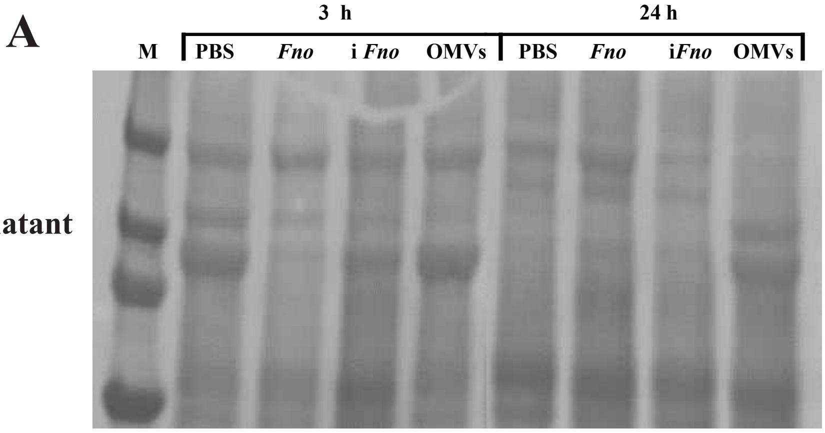

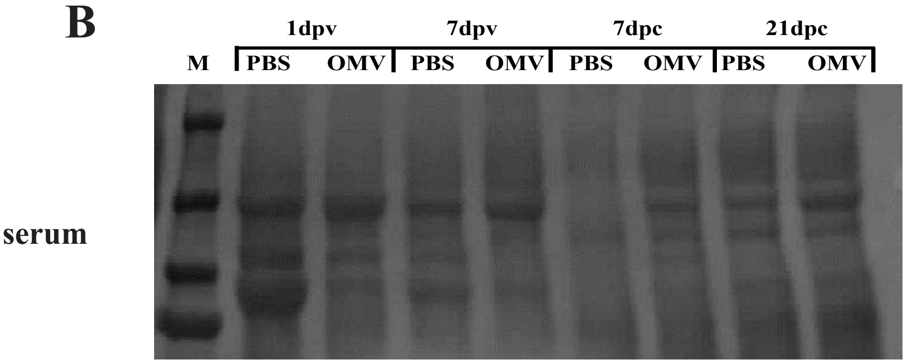

Figure 3S. Representative Western blot membrane showing protein content. **A.** Ponceau staining of supernatant protein after transfer to a membrane. Primary Zebrafish kidney cells cultivated *in vitro* for 3 h and 24 h in presence of PBS, *Fno* (1x10^8 CFU/ml), *Fno* inactivated in formalin (iFno) (1x10^8 CFU/ml) or MVs (20µg). **B.** Ponceau staining of serum protein of zebrafish injected with PBS or 20 µg MVs at 1 or 7 day post vaccination (dpv); 7 or 21 days post challenge (dpc) with *Fno* (1x10^6 CFU) after transfer to a membrane. M: marker in kilo Daltons (kDa). The same amount of protein was load in each lane as measured by nanodrop, but different protein bands were observed between the different treatment and time point.

Table 1S. Primers used in qRT-PCR analysis

Gene		Sequence 5' - 3'	Amplicon	Access number	Gene ID	Gen Name ZFIN
18s	F	ACCACCCACAGAATCGAGAAA	98	FJ915075.1	100037361	*zgc:158463*
	R	GCCTGCGGCTTAATTTGACT				
ef1α	F	CTTCTCAGGCTGACTGTGC	142	FJ915061.1	30516	*eef1a1l1*
	R	CCGCTAGCATTACCCTCC				
il-1β	F	TTCCCCAAGTGCTGCTTATT	149	NM_212844.2	405770	*il1b*
	R	AAGTTAAAACCGCTGTGGTCA				
mpx	F	TGATGTTTGGTTAGGAGGTG	101	BC068379.1	337514	*mpx*
	R	GAGCTGTTTTCTGTTTGGTG				
mpeg	F	TACAGCACGGGTTCAAGTCCGT	187	NM_212737	335407	*mpeg1.1*
	R	ACTTGTGATGACATGGGTGCCG				
mhcii	F	TGACTCAACTGTCCGTGATA	195	BC083466.1	449770	*zgc:103700*
	R	CCATTAGCCATCTCCATAGTG				
igm	F	GAAGCCTCCAATTCTGTTGG	147	AY643751	378762	*ighm*
	R	CCGGGTAAACACATGAAG				
tnf-α1	F	GGTCCTCTGTTGCGGAGGCG	108	NM_212859	405785	*tnfa*
	R	CGAGCTGGAAGTCGCCCTGAG				
tnf-α2	F	GAGCAAATCCGTTATTCTCG	95	NM_001024447	554167	*tnfb*
	R	GAGGGTGATGTCTGATAGAAC				
igd	F	GACACATTAGCCCATCAGCA	156	BX510335	101883016	*ighd*
	R	CTGGAGAGCAGCAAAAGGAT				
igz/t	F	GAACCAAACTCAGGGTTGGA	152	AY643750	446139	*ighz*
	R	CACCCAGCATTCTACAGCAA				
il-12p35		QT02085300	QUIAGEN	NM_001007107	445410	*il12a*
ifn-γ1-1		QT02151961	QUIAGEN	NM_001020793	553965	*ifng1-1*
ifn-γ1-2		QT02064328	QUIAGEN	NM_212864	405790	*ifng1-2*
ifnphi1		QT02223109	QUIAGEN	NM_207640	360134	*ifnphi1*
ifnphi2		QT02183839	QUIAGEN	NM_001111082	100126802	*ifnphi2*
ifnphi3(1)		QT02139256	QUIAGEN	NM_001111083	100126803	*ifnphi3*
ifnphi3(2)		QT02139263	QUIAGEN	NM_001111083	100126803	*ifnphi3*
mx-a		QT02179205	QUIAGEN	NM_182942	360142	*mxa*

Table 2S.

Proteins Identified in OMVs from *Francisella noatunensis* subsp. *orientalis*

Protein	Peptide hits	Protein product	Gene locus	Predicted subcellular location
Bifunctional proline dehydrogenase/pyrroline-5-carboxylate	158	WP_014715611.1	OOM_1728	Cytoplasmic
Intracellular growth locus, subunit C	101	WP_014714932.1	OOM_0933	Unknown
60kDa chaperonin GroEL	92	WP_014714726.1	OOM_0688	Cytoplasmic
ATP synthase subunit b	91	WP_004286980.1	OOM_0555	CytoplasmicMembrane
ATP synthase subunit alpha	89	WP_014714619.1	OOM_0557	Cytoplasmic
Outer membrane associated protein	88	WP_014715233.1	OOM_1284	OuterMembrane
Catalase-peroxidase	81	WP_014715162.1	OOM_1206	Unknown/ multiple localization
OmpA family protein	76	WP_014714420.1	OOM_0329	OuterMembrane
Succinate dehydrogenase iron-sulfur subunit	75	WP_014714631.1	OOM_0570	CytoplasmicMembrane
Intracellular growth locus protein B	73	WP_014714931.1	OOM_0932	Cytoplasmic
Putative uncharacterized protein	63	WP_014714929.1	OOM_0930	OuterMembrane
Ribonuclease E	59	WP_014715484.1	OOM_1576	Cytoplasmic
Pyruvate dehydrogenase E1 component	58	WP_014714759.1	OOM_0731	Cytoplasmic
Serine-type D-Ala-D-Ala carboxypeptidase	56	WP_014714959.1	OOM_0962	Unknown/ multiple localization
Chaperone ClpB	53	WP_014715663.1	OOM_1793	Cytoplasmic
Glycerol-3-phosphate dehydrogenase	51	WP_014714672.1	OOM_0621	Unknown/ multiple localization
Enolase	51	WP_014715152.1	OOM_1196	Cytoplasmic
Succinate dehydrogenase	48	WP_014714630.1	OOM_0569	CytoplasmicMembrane
Peptidyl-prolyl cis-trans isomerase	46	WP_014715675.1	OOM_1806	OuterMembrane
ATP synthase gamma chain	45	WP_014714620.1	OOM_0558	Cytoplasmic
HflK-HflC membrane protein complex, HflK	43	WP_014715271.1	OOM_1329	Cytoplasmic
tRNA adenylyltransferase	43	WP_014714748.1	OOM_0718	Cytoplasmic
Chaperone protein DnaK	42	WP_014714552.1	OOM_0476	Cytoplasmic
Polyribonucleotide nucleotidyltransferase	41	WP_014715145.1	OOM_1186	Cytoplasmic
microtubule-severing ATPase	41	WP_014715206.1	OOM_1255	CytoplasmicMembrane
dTDP-4-dehydrorhamnose 3,5-epimerase	40	WP_014714804.1	OOM_0799	Unknown
MMS1, Mono-functional DNA-alkylating agent methyl	39	WP_014714941.1	OOM_0943	Unknown/ multiple localization
septum formation inhibitor-activating ATPase	38	WP_014714409.1	OOM_0313	Cytoplasmic
Cell division protein FtsZ	38	WP_014714255.1	OOM_0142	Cytoplasmic
Putative uncharacterized protein	36	WP_014715133.1	OOM_1173	CytoplasmicMembrane
Putative uncharacterized protein	36	WP_014714436.1	OOM_0347	Unknown

Table 2S.

50S ribosomal protein L2	35	WP_014714331.1	OOM_0224	Cytoplasmic
LemA-like protein	35	WP_014714465.1	OOM_0377	Cytoplasmic
Peptidyl-prolyl cis-trans isomerase	33	WP_014714970.1	OOM_0973	OuterMembrane
Protein TolB	32	WP_014714429.1	OOM_0338	Periplasmic
30S ribosomal protein S1	32	WP_014714250.1	OOM_0137	Cytoplasmic
dihydrolipoamide acetyltransferase	32	WP_014714760.1	OOM_0732	Cytoplasmic
Succinyl-CoA ligase [ADP-forming] subunit beta	31	WP_014715132.1	OOM_1172	Cytoplasmic
Cell entry related family protein	31	WP_014714564.1	OOM_0490	Unknown
30S ribosomal protein S5	31	WP_014714344.1	OOM_0238	Cytoplasmic
2-oxoglutarate dehydrogenase complex, E2 component	31	WP_014714633.1	OOM_0572	Cytoplasmic
Putative uncharacterized protein	31	WP_014714497.1	OOM_0414	Unknown
ATP synthase subunit beta	30	WP_014714621.1	OOM_0559	Cytoplasmic
Elongation factor Tu	30	WP_014714676.1	OOM_0626	Cytoplasmic
Elongation factor G	30	WP_014714327.1	OOM_0219	Cytoplasmic
DsbA_Com1_like, DsbA family, Com1-like subfamily	30	WP_014714996.1	OOM_1001	Unknown
Succinyl-CoA ligase [ADP-forming] subunit alpha	30	WP_014715131.1	OOM_1171	Cytoplasmic
50S ribosomal protein L21	30	WP_014715213.1	OOM_1262	Cytoplasmic
Putative uncharacterized protein	29	WP_014714940.1	OOM_0942	Unknown
Membrane fusion protein	29	WP_014714557.1	OOM_0482	Unknown/ multiple localization
Cytochrome d ubiquinol oxidase subunit I	29	WP_014714285.1	OOM_0172	CytoplasmicMembrane
Membrane fusion protein	28	WP_014714655.1	OOM_0599	CytoplasmicMembrane
ATP-dependent protease ATP-binding subunit	27	WP_014715311.1	OOM_1378	Cytoplasmic
VacJ like lipoprotein	27	WP_014714402.1	OOM_0305	OuterMembrane
Glutamate dehydrogenase	27	WP_014714731.1	OOM_0696	Unknown/ multiple localization
OmpA family peptidoglycan-associated lipoprotein	25	WP_014714431.1	OOM_0340	OuterMembrane
RmuC family protein	24	WP_014715293.1	OOM_1356	Cytoplasmic
Putative uncharacterized protein	24	WP_014714891.1	OOM_0882	Unknown
Peptidyl-prolyl cis-trans isomerase SurA	24	WP_014715101.1	OOM_1137	Periplasmic
Protein-export membrane protein SecF	24	WP_014715251.1	OOM_1307	CytoplasmicMembrane
NADH dehydrogenase (Quinone)	24	WP_014714596.1	OOM_0531	Cytoplasmic
Putative uncharacterized protein	24	WP_014715252.1	OOM_1308	Unknown
Acetyl-CoA carboxylase, biotin carboxy carrier protein subunit	24	WP_014715105.1	OOM_1141	Unknown
Glycerol-3-phosphate acyltransferase	23	WP_014715244.1	OOM_1297	CytoplasmicMembrane
Putative uncharacterized protein	23	WP_014714184.1	OOM_0066	Cytoplasmic

Table 2S.

GDP-mannose 4,6-dehydratase	21	WP_014714812.1	OOM_0777	Cytoplasmic
Toluene tolerance	21	WP_014714405.1	OOM_0308	Unknown
Heat shock protein HtpG	21	WP_014714354.1	OOM_0249	Cytoplasmic
Putative uncharacterized protein	21	WP_014714496.1	OOM_0413	OuterMembrane
NADH-quinone oxidoreductase subunit C	21	WP_014714594.1	OOM_0529	Cytoplasmic
Putative cyanophycin synthetase	20	WP_014715239.1	OOM_1291	Cytoplasmic
DNA-directed RNA polymerase subunit beta	20	WP_014714682.1	OOM_0633	Cytoplasmic
Cell division protein	20	WP_014714384.1	OOM_0280	CytoplasmicMembrane
Putative uncharacterized protein	20	WP_014714203.1	OOM_0085	Unknown/ multiple localization
Ubiquinone/menaquinone biosynthesis C-methyltransferase UbiE	20	WP_014714522.1	OOM_0442	Cytoplasmic
Lipid A export ATP-binding/permease protein MsbA	20	WP_014714657.1	OOM_0602	CytoplasmicMembrane
50S ribosomal protein L9	20	WP_014714979.1	OOM_0983	Cytoplasmic
Phosphatidylserine decarboxylase proenzyme	20	WP_014715025.1	OOM_1043	CytoplasmicMembrane
Putative uncharacterized protein	20	WP_014714412.1	OOM_0317	Unknown
NADH dehydrogenase	20	WP_014715682.1	OOM_1816	Cytoplasmic
Protein translocase subunit SecD	19	WP_014715250.1	OOM_1306	CytoplasmicMembrane
AMP-binding protein	19	WP_014715140.1	OOM_1181	CytoplasmicMembrane
Putative glutamate synthase	19	WP_014714897.1	OOM_0890	Cytoplasmic
Putative lipoprotein	19	WP_014714379.1	OOM_0274	Unknown
Peptide methionine sulfoxide reductase MsrA	19	WP_014715695.1	OOM_1832	Unknown
50S ribosomal protein L17	19	WP_014714352.1	OOM_0247	Cytoplasmic
Putative uncharacterized protein	18	WP_014714796.1	OOM_0779	OuterMembrane
Alpha-ketoglutarate decarboxylase	18	WP_014714632.1	OOM_0571	Cytoplasmic
30S ribosomal protein S11	18	WP_003021583.1	OOM_0244	Cytoplasmic
Pyridoxal biosynthesis lyase PdxS	18	WP_014715137.1	OOM_1178	Cytoplasmic
Lipoprotein	18	WP_014715467.1	OOM_1553	Unknown
DNA-binding protein HU-beta	18	WP_014715264.1	OOM_1322	Cytoplasmic
NADH dehydrogenase subunit G	17	WP_014714597.1	OOM_0532	Cytoplasmic
tRNA nucleotidyltransferase	17	WP_014714461.1	OOM_0373	Cytoplasmic
Putative uncharacterized protein	17	WP_014714563.1	OOM_0489	Unknown
Putative uncharacterized protein	17	WP_014715220.1	OOM_1271	Cytoplasmic
ATP synthase epsilon chain	17	WP_014714622.1	OOM_0560	Unknown/ multiple localization
Cell wall biosynthesis glycosyltransferase-like protein	17	WP_014714838.1	OOM_0822	Cytoplasmic

Table 2S.

Soluble lytic murein transglycosylase	16	WP_014715037.1	OOM_1057	Periplasmic
NADH-quinone oxidoreductase subunit I	16	WP_012280348.1	OOM_0534	Cytoplasmic
Glycerophosphoryl diester phosphodiesterase	16	WP_014715164.1	OOM_1209	Periplasmic
Protein HflC	16	WP_014715272.1	OOM_1330	Cytoplasmic
50S ribosomal protein L7/L12	16	WP_014714681.1	OOM_0632	Unknown/ multiple localization
pyruvate dehydrogenase complex, E3 component, dihydrolipoamide	16	WP_014714761.1	OOM_0733	Cytoplasmic
30S ribosomal protein S3	16	WP_014714334.1	OOM_0227	Cytoplasmic
30S ribosomal protein S7	16	WP_014714326.1	OOM_0218	Cytoplasmic
Phosphopentomutase	16	WP_014714661.1	OOM_0606	Cytoplasmic
ATP synthase subunit C	16	WP_014714617.1	OOM_0554	CytoplasmicMembrane
Protein-L-isoaspartate O-methyltransferase	15	WP_014714573.1	OOM_0501	Cytoplasmic
exoribonuclease II	15	WP_014714788.1	OOM_0764	Cytoplasmic
Elongation factor Ts	15	WP_014714319.1	OOM_0210	Cytoplasmic
ATP-binding cassette (ABC) supeorfamily protein	15	WP_014714869.1	OOM_0854	CytoplasmicMembrane
50S ribosomal protein L16	15	WP_014714335.1	OOM_0228	Cytoplasmic
Acyltransferase	15	WP_014715669.1	OOM_1800	CytoplasmicMembrane
Putative uncharacterized protein	15	WP_014715657.1	OOM_1786	Unknown
2-nonaprenyl-3-methyl-6-methoxy-1,4-benzoquinol hydroxylase	15	WP_014715635.1	OOM_1757	Cytoplasmic
Glyceraldehyde-3-phosphate dehydrogenase (Phosphorylating)	14	WP_014714923.1	OOM_0922	Cytoplasmic
Membrane protein	14	WP_014715198.1	OOM_1246	CytoplasmicMembrane
Heat shock protein HtpX	14	WP_014714464.1	OOM_0376	CytoplasmicMembrane
Glycosyl transferase group 1 family protein	14	WP_014715478.1	OOM_1569	Cytoplasmic
Two-component regulator, sensor histidine kinase	14	WP_014714646.1	OOM_0590	CytoplasmicMembrane
ABC transporter, ATP-binding protein	14	WP_014714236.1	OOM_0120	CytoplasmicMembrane
Putative uncharacterized protein	14	WP_014714498.1	OOM_0416	OuterMembrane
30S ribosomal protein S12	14	WP_003035357.1	OOM_0217	Cytoplasmic
Cell division topological specificity factor	14	WP_012279974.1	OOM_0312	Cytoplasmic
Co/Zn/Cd cation transporter	14	WP_014715336.1	OOM_1404	CytoplasmicMembrane
30S ribosomal protein S6	14	WP_013923199.1	OOM_0985	Cytoplasmic
malate dehydrogenase	13	WP_014715200.1	OOM_1248	Cytoplasmic
Serine hydroxymethyltransferase	13	WP_014715473.1	OOM_1564	Cytoplasmic
Serine-type D-Ala-D-Ala carboxypeptidase	13	WP_014714967.1	OOM_0970	Unknown/ multiple localization

Table 2S.

Cell division protein ftsA	13	WP_014714254.1	OOM_0141	Cytoplasmic
Fructose-1,6-bisphosphate aldolase	13	WP_014714925.1	OOM_0925	Cytoplasmic
30S ribosomal protein S2	13	WP_014714318.1	OOM_0209	Cytoplasmic
Preprotein translocase family protein	13	WP_014715249.1	OOM_1305	CytoplasmicMembrane
Heavy metal cation transport ATPase	13	WP_014714468.1	OOM_0380	CytoplasmicMembrane
2', 3'-cyclic nucleotide 2'-phosphodiesterase	13	WP_014715225.1	OOM_1276	Periplasmic
Translation initiation factor IF-2	12	WP_014714608.1	OOM_0545	Cytoplasmic
enoyl-ACP reductase I	12	WP_014715506.1	OOM_1600	CytoplasmicMembrane
DNA-directed RNA polymerase subunit beta	12	WP_014714683.1	OOM_0634	Cytoplasmic
30S ribosomal protein S10	12	WP_004286618.1	OOM_0220	Cytoplasmic
50S ribosomal protein L1	12	WP_014714679.1	OOM_0630	Cytoplasmic
Acyl carrier protein	12	WP_004287370.1	OOM_0914	Cytoplasmic
2-alkenal reductase	12	WP_014715581.1	OOM_1695	CytoplasmicMembrane
Ferrochelatase	12	WP_014715615.1	OOM_1735	Cytoplasmic
Peptidase	12	WP_014715529.1	OOM_1628	CytoplasmicMembrane
Phosphorylase	11	WP_014715058.1	OOM_1084	Cytoplasmic
50S ribosomal protein L10	11	WP_014714680.1	OOM_0631	Cytoplasmic
Chaperone protein DnaJ	11	WP_014714553.1	OOM_0477	Cytoplasmic
50S ribosomal protein L20	11	WP_014715541.1	OOM_1643	Cytoplasmic
Single-stranded DNA-binding protein	11	WP_014714219.1	OOM_0102	Cytoplasmic
beta-hydroxyacyl-ACP dehydratase	11	WP_014714772.1	OOM_0745	Cytoplasmic
Acyltransferase family	11	WP_014714808.1	OOM_0808	Cytoplasmic
septum formation inhibitor	11	WP_014714410.1	OOM_0314	Cytoplasmic
ABC transporter, ATP-binding protein	11	WP_014714954.1	OOM_0957	Cytoplasmic
Putative uncharacterized protein	11	WP_014715280.1	OOM_1342	CytoplasmicMembrane
Ferric iron binding protein, ferritin-like protein	11	WP_014715286.1	OOM_1349	Cytoplasmic
Protein translocase subunit SecY	11	WP_014714347.1	OOM_0241	CytoplasmicMembrane
Putative uncharacterized protein	11	WP_014714382.1	OOM_0278	CytoplasmicMembrane
50S ribosomal protein L3	10	WP_014714328.1	OOM_0221	Cytoplasmic
competence lipoprotein ComL	10	WP_014714569.1	OOM_0495	OuterMembrane
Phosphoenolpyruvate carboxylase	10	WP_014715088.1	OOM_1118	Cytoplasmic
Biopolymer transport protein	10	WP_014714427.1	OOM_0336	CytoplasmicMembrane
50S ribosomal protein L28	10	WP_004286518.1	OOM_0316	Cytoplasmic
Major Facilitator Superfamily	10	WP_014714653.1	OOM_0597	CytoplasmicMembrane

Table 2S.

RgpF, Rhamnan synthesis protein F	10	WP_014714845.1	OOM_0829	Cytoplasmic
DEAD/DEAH box helicase	9	WP_014715362.1	OOM_1434	Cytoplasmic
Dihydrolipoamide dehydrogenase	9	WP_014714734.1	OOM_0702	Cytoplasmic
Polyamine transporter, ABC transporter,ATP-binding protein	9	WP_014715217.1	OOM_1267	CytoplasmicMembrane
Acetyl-CoA carboxylase, biotin carboxylase subunit	9	WP_014715106.1	OOM_1142	Cytoplasmic
ABC transporter ATPase	9	WP_014715044.1	OOM_1067	CytoplasmicMembrane
50S ribosomal protein L5	9	WP_014714340.1	OOM_0233	Cytoplasmic
Putative uncharacterized protein	9	WP_014715555.1	OOM_1658	Cytoplasmic
ABC transporter, ATP-binding protein	9	WP_014714408.1	OOM_0311	CytoplasmicMembrane
Membrane protein insertase YidC	9	WP_014714168.1	OOM_0048	CytoplasmicMembrane
Pseudouridine synthase	9	WP_014714976.1	OOM_0979	Cytoplasmic
Intracellular growth locus protein D	9	WP_014714933.1	OOM_0934	Cytoplasmic
NADH dehydrogenase (Quinone)	9	WP_014714602.1	OOM_0538	CytoplasmicMembrane
50S ribosomal protein L14	9	WP_014714338.1	OOM_0231	Cytoplasmic
Putative uncharacterized protein	9	WP_014715165.1	OOM_1210	Unknown
leucyl aminopeptidase	9	WP_014715199.1	OOM_1247	Cytoplasmic
Putative uncharacterized protein	8	WP_014714585.1	OOM_0514	Unknown
Cell division protein FtsI/penicillin-binding protein	8	WP_014715143.1	OOM_1184	Unknown/ multiple localization
HlyD family membrane fusion protein	8	WP_014714151.1	OOM_0023	CytoplasmicMembrane
Putative uncharacterized protein	8	WP_014714511.1	OOM_0430	Unknown/ multiple localization
Stearoyl-CoA 9-desaturase	8	WP_014714685.1	OOM_0637	CytoplasmicMembrane
O-antigen export system ATP-binding protein orfbB	7	WP_014714849.1	OOM_0833	CytoplasmicMembrane
GTP-binding protein EngA	7	WP_014714513.1	OOM_0432	CytoplasmicMembrane
50S ribosomal protein L11	7	WP_012280431.1	OOM_0629	Cytoplasmic
ABC transporter	7	WP_014715043.1	OOM_1066	CytoplasmicMembrane
Lysophospholipase_L1_like, Lysophospholipase L1-like protein	7	WP_014714949.1	OOM_0951	Periplasmic
host factor I for bacteriophage Q beta replication	7	WP_014715267.1	OOM_1325	Cytoplasmic
UDP-3-O-acylglucosamine N-acyltransferase	7	WP_014714771.1	OOM_0744	Cytoplasmic
aspartyl-tRNA synthetase	7	WP_014714225.1	OOM_0109	Cytoplasmic
Putative lipoprotein	7	WP_014714555.1	OOM_0479	Unknown
Sugar_tr, Sugar (And other) transporter	7	WP_014715361.1	OOM_1431	CytoplasmicMembrane
M24 family peptidase	7	WP_014715585.1	OOM_1700	Cytoplasmic
Signal peptidase I	7	WP_014714786.1	OOM_0761	CytoplasmicMembrane
Chromosomal replication initiator protein DnaA	7	WP_014714132.1	OOM_0001	Cytoplasmic

Table 2S.

Sel1-like protein	7	WP_014714370.1	OOM_0265	Unknown
type IV pilus prepilin peptidase PilD	7	WP_014715308.1	OOM_1374	CytoplasmicMembrane
Arsenate reductase	7	WP_014714800.1	OOM_0789	Cytoplasmic
Signal recognition particle receptor FtsY	6	WP_014714665.1	OOM_0612	Cytoplasmic
Carbonic anhydrase	6	WP_014715258.1	OOM_1314	Cytoplasmic
Ferrous iron transport protein B	6	WP_014714724.1	OOM_0684	CytoplasmicMembrane
Phosphoglucosamine mutase	6	WP_014714635.1	OOM_0574	Cytoplasmic
ATP-dependent protease peptidase subunit	6	WP_014715312.1	OOM_1379	Cytoplasmic
Cysteine/glutathione ABC transporter membrane/ATP-binding component	6	WP_014715178.1	OOM_1225	CytoplasmicMembrane
Pathogenicity determinant protein pdpA1	6	WP_014714942.1	OOM_0944	Unknown/ multiple localization
Glutathione synthetase	6	WP_014715412.1	OOM_1486	Cytoplasmic
Membrane-bound lytic murein transglycosylase	6	WP_014714550.1	OOM_0474	Unknown/ multiple localization
Homoserine/threonine efflux family protein	6	WP_014714873.1	OOM_0858	CytoplasmicMembrane
NADH-quinone oxidoreductase subunit A	6	WP_014714593.1	OOM_0527	CytoplasmicMembrane
Putative uncharacterized protein	6	WP_014715019.1	OOM_1037	Extracellular
Putative uncharacterized protein	6	WP_014714364.1	OOM_0259	CytoplasmicMembrane
Type IV pili, pilus assembly protein	6	WP_014714664.1	OOM_0611	Unknown
Glycosyl transferase family protein	6	WP_014714851.1	OOM_0835	Unknown
Putative uncharacterized protein	6	WP_014715026.1	OOM_1044	Unknown
30S ribosomal protein S16	6	WP_004287066.1	OOM_0639	Cytoplasmic
Glycosyltransferase	6	WP_014714711.1	OOM_0670	Unknown
Putative capsule biosynthesis protein	6	WP_014715532.1	OOM_1631	CytoplasmicMembrane
Putative uncharacterized protein	6	WP_014714937.1	OOM_0938	Unknown
Aminomethyltransferase	6	WP_014715046.1	OOM_1069	Cytoplasmic
Transketolase	6	WP_014714935.1	OOM_0936	Cytoplasmic
ornithine cyclodeaminase	6	WP_014714814.1	OOM_0780	Cytoplasmic

Table 3S. Ct values from the RT-qPCR data presented in Figure 6.

	1dpv		7dpv		21dpv		1dpc		7dpc	
	**PBS**	**MVs**	**PBS**	**MVs**	**PBS**	**MVs**	**PBS**	**MVs**	**PBS**	**MVs**
zgc:158463	15,6 ± 0,9	15,6 ± 0,4	16,2 ± 0,6	15,7 ± 0,3	15,6 ± 0,2	16,6 ± 1,8	15,6 ± 1,3	16,6 ± 0,7	15,4 ± 0,5	14,8 ± 0,8
ifnphi1	25,4 ± 0,5	26,4 ± 0,9	27,7 ± 1	26,6 ± 0,8	26 ± 0,7	24,8 ± 0,1	25,4 ± 0,5	26,1 ± 0,3	24,6 ± 0,4	24,3 ± 0,5
ifnphi2	31,7 ± 0,5	32,5 ± 0,9	31,7 ± 0,4	31,7 ± 0,2	32,4 ± 0,6	30,4 ± 0,7	31,7 ± 0,5	30,9 ± 0,4	29 ± 0,8	29,2 ± 0,5
ifnphi3(1)	31,6 ± 0,4	33,9 ± 0,8	33,7 ± 0,7	33,2 ± 1,1	33,4 ± 0,6	31,1 ± 0,5	31,6 ± 0,4	30,8 ± 0,7	29,6 ± 0,9	29,6 ± 0,8
ifnphi3(2)	31,6 ± 0,4	32,1 ± 0,4	34 ± 0,2	33,2 ± 0,6	33,1 ± 0,1	30,6 ± 0,1	31,6 ± 0,4	31,5 ± 0,9	29,3 ± 0,8	29 ± 0,9
mxa	28,1 ± 0,9	26,8 ± 1,7	30,7 ± 0,5	29,4 ± 0,3	30,4 ± 0,7	28,8 ± 1,8	28,1 ± 1,3	28,6 ± 1,8	26,8 ± 0,3	24,8 ± 1,7
tnfa	22,6 ± 0,7	24,9 ± 0,3	22,8 ± 0,6	23,2 ± 0,3	25,4 ± 0,1	24,2 ± 0,3	22,6 ± 0,7	24,4 ± 0,3	22,6 ± 0,5	22,3 ± 0,5
tnfb	37,5 ± 0,8	40,6 ± 2,4	25,4 ± 1	37,7 ± 1,2	41 ± 0,6	41 ± 0,7	37,5 ± 1,3	40 ± 0,7	37,5 ± 0,9	36,4 ± 1,8
ifng1-1	28,7 ± 0,7	24,5 ± 1,3	30,4 ± 0,9	31,4 ± 0,6	34,7 ± 0,7	29,4 ± 2,4	28,7 ± 1,4	32,1 ± 1	28,7 ± 0,9	29,5 ± 0,7
ifng1-2	23 ± 0,7	24,2 ± 0,8	24,1 ± 0,5	24,8 ± 1	27,6 ± 0,8	24,1 ± 0,4	23 ± 0,7	24,9 ± 0,8	23 ± 0,5	23 ± 0,6
il12a	32,2 ± 0,2	29,7 ± 0,5	32,2 ± 0,7	32,5 ± 0,1	31 ± 0,2	31,8 ± 0,1	32,2 ± 0,2	31,6 ± 0,2	30 ± 0,2	30,3 ± 0,4
il1b	26,6 ± 0,9	23,3 ± 0,9	26,3 ± 0,6	24 ± 0,6	27,7 ± 0,5	25 ± 0,2	26,6 ± 0,9	23,9 ± 0,6	23,1 ± 0,6	22,3 ± 0,3
